# Evolution of Listeria monocytogenes in a Food Processing Plant Involves Limited Single-Nucleotide Substitutions but Considerable Diversification by Gain and Loss of Prophages

**DOI:** 10.1128/AEM.02493-19

**Published:** 2020-03-02

**Authors:** Anna Sophia Harrand, Balamurugan Jagadeesan, Leen Baert, Martin Wiedmann, Renato H. Orsi

**Affiliations:** a Department of Food Science, Cornell University, Ithaca, New York, USA; b Nestlé Institute of Food Safety and Analytical Sciences, Nestlé Research, Vers-chez-les-Blanc, Lausanne, Switzerland; The Pennsylvania State University

**Keywords:** attachment, *Listeria monocytogenes*, sanitizer, bacteriophages, evolution, food processing facility, persistence, plasmids, smoked salmon, whole-genome sequencing

## Abstract

Whole-genome sequencing (WGS) is becoming the standard method for subtyping Listeria monocytogenes. Interpretation of WGS data for isolates from foods and associated environments is, however, challenging due to a lack of detailed data on *Listeria* evolution in processing facilities. Here, we used previously collected WGS data for 40 L. monocytogenes isolates obtained from a cold-smoked salmon processing facility between 1998 and 2015 to probe the L. monocytogenes molecular evolution in this facility, combined with phenotypic assessment of selected isolates. Isolates represented three clusters (1, 2, and 3); cluster 3 isolates (*n* = 32) were obtained over 18 years. The average mutation rate for cluster 3 was estimated as 1.15 × 10^−7^ changes per nucleotide per year (∼0.35 changes per genome per year); the most recent common ancestors (MRCAs) of subclusters 3a and 3b were estimated to have occurred around 1958 and 1974, respectively, within the age of the facility, suggesting long-term persistence in this facility. Extensive prophage diversity was observed within subclusters 3a and 3b, which have one shared and six unique prophage profiles for each subcluster (with 16 prophage profiles found among all 40 isolates). The plasmid-borne sanitizer tolerance operon *bcrABC* was found in all cluster 2 and 3 isolates, while the transposon-borne sanitizer tolerance gene *qacH* was found in one cluster 1 isolate; presence of these genes was correlated with the ability to survive increased concentrations of sanitizers. Selected isolates showed significant variation in the ability to attach to surfaces, with persistent isolates attaching better than transient isolates at 21°C.

**IMPORTANCE** Knowledge about the genetic evolution of L. monocytogenes in food processing facilities over multiple years is generally lacking. This information is critical to interpret WGS findings involving food or food-associated isolates. This study suggests that L. monocytogenes that persists in processing facilities may evolve with a low single-nucleotide mutation rate mostly driven by negative (i.e., purifying) selection but with rapid diversification of prophages. Hence, isolation of L. monocytogenes with few single-nucleotide polymorphism (SNP) differences in different locations (e.g., supplier plants and receiving plants) is possible, highlighting the importance of epidemiological and detailed isolate metadata for interpreting WGS data in traceback investigation. Our study also shows how advanced WGS data analyses can be used to support root cause analysis efforts and may, for example, pinpoint the time when a persistence event started (which then potentially could be linked to facility changes, introduction of new equipment, etc.).

## Abstract (precis)

Knowledge about the genetic evolution of L. monocytogenes in food processing facilities over multiple years is generally lacking. This information is critical to interpret WGS findings involving food or food-associated isolates. This study suggests that L. monocytogenes that persists in processing facilities may evolve with a low single-nucleotide mutation rate mostly driven by negative (i.e., purifying) selection but with rapid diversification of prophages. Hence, isolation of L. monocytogenes with few single-nucleotide polymorphism (SNP) differences in different locations (e.g., supplier plants and receiving plants) is possible, highlighting the importance of epidemiological and detailed isolate metadata for interpreting WGS data in traceback investigation. Our study also shows how advanced WGS data analyses can be used to support root cause analysis efforts and may, for example, pinpoint the time when a persistence event started (which then potentially could be linked to facility changes, introduction of new equipment, etc.).

## INTRODUCTION

Listeria monocytogenes strains can persist in food processing facilities over a long period of time (more than 10 years) (1–3). Among food categories prone to L. monocytogenes contamination, ready-to-eat (RTE) foods are of particular concern as no kill step is expected to be applied before food consumption. Cold-smoked fish production presents a challenge to the industry as no heating step is applied in any step of the process. Across food categories, L. monocytogenes persistence represents a concern for processors; therefore, tools and data to identify persistence are essential. As whole-genome sequencing (WGS) becomes the standard tool for L. monocytogenes subtyping, understanding the rate of evolution of isolates, at the genome level, in a facility is crucial for an effective investigation. More specifically, a better understanding of the evolutionary rate in food processing facilities will allow improved interpretation of observed SNP differences as our data will provide new information on time frames that typically lead to an observed number of single-nucleotide polymorphism (SNP) differences. In addition, persistent isolates may harbor genetic features that allow better survival and growth in food processing environments, such as sanitizer and stress resistance genes (4, 5). These genes are often found on mobile elements that facilitate their spread through horizontal gene transfer across a population.

**FIG 1:**
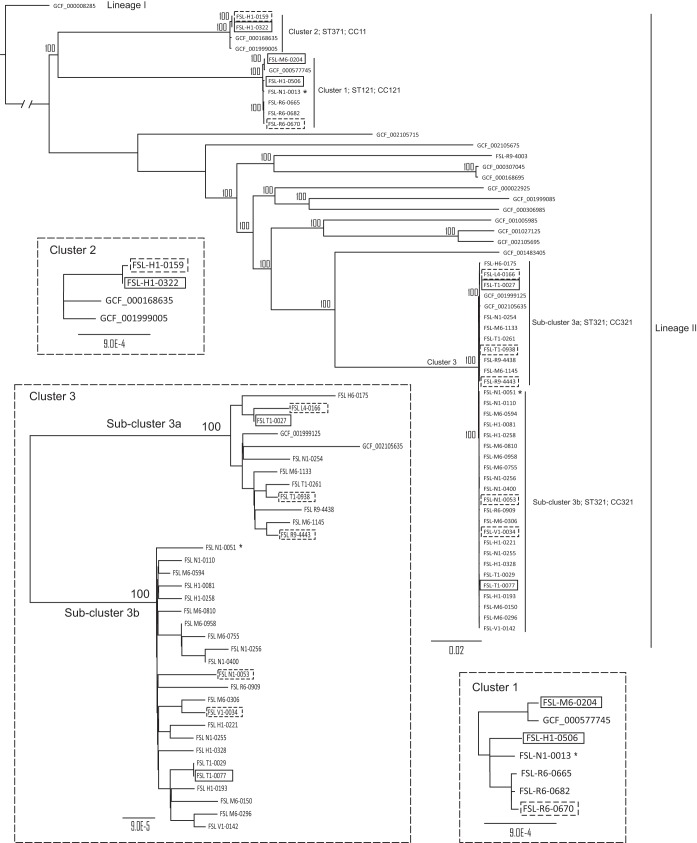
Reference-free k-mer-based phylogenetic tree. Core SNPs identified using kSNP3 among the 42 isolates analyzed in this study plus 17 L. monocytogenes genomes downloaded from NCBI were used to construct this maximum likelihood tree. Branch lengths are proportional to the genetic distance between nodes or isolates. Bootstrap (*n* = 1,000) values are shown on top of the major branches. Clusters and subclusters described in Jagadeesan et al. (6), their respective sequence types (ST), and clonal complexes (CC) are annotated. An asterisk (*) after the isolate name indicates an isolate not collected in facility X. Solid boxes indicate isolates that were used for the phenotypic experiments involving (i) reduced sensitivity to four sanitizers, (ii) attachment to an abiotic surface, (iii) and growth under stress conditions. Dashed boxes indicate additional isolates added to the experiments involving (i) reduced sensitivity to the sanitizer BC and (ii) attachment to an abiotic surface. The lineages of the sequences are also annotated. The three insets show detailed depictions of the relationship within clusters 1, 2, and 3 (please note the different scales below the trees).

In this study, we analyzed a set of 40 L. monocytogenes isolates collected over 18 years from a single cold-smoked salmon processing facility (facility X), along with 2 isolates collected from two other cold-smoked salmon processing facilities, for a total of 42 isolates. We have previously completed whole-genome sequencing of these 42 isolates. Reference-free SNP analysis and seven-gene multilocus sequence typing (MLST) previously allowed classification of the 42 isolates into three distinct clusters, namely, cluster 1 (*n* = 6; sequence type 121 [ST121]; clonal complex 121 [CC121]), cluster 2 (*n* = 2; ST371; CC11), and cluster 3 (*n* = 33; ST321; CC321), in addition to one unclustered isolate (ST199; CC199) (6). Among the 40 facility X isolates, most isolates (*n* = 32) were classified into cluster 3, which could be further divided into two subclusters, designated 3a and 3b (Fig. 1). In addition, high-quality SNP (hqSNP) analysis was used to assess the relationship between isolates within a cluster. Facility X isolates from cluster 1 were previously shown to represent (i) three isolates with <10 hqSNP differences from each other, collected in the same month, suggesting a single introduction event, and (ii) two genetically distinct isolates with >50 hqSNP differences from each other and any other isolates in this cluster (6). The two isolates classified into cluster 2 previously showed <10 hqSNP differences from each other but were collected in the same year (4 months apart), suggesting a short period of persistence in the facility (6). On the other hand, the 10 isolates from facility X classified into subcluster 3a and the 22 isolates classified into subcluster 3b previously showed <50 hqSNP differences from other isolates classified into the same subcluster, including a pair of subcluster 3b isolates that showed no hqSNP differences; isolates in each subcluster were collected over a period of >10 years (6). Although this close relatedness over several years suggests that the subcluster 3a and 3b isolates persisted and diversified within the facility, multiple reintroductions of some of the isolates from an external source cannot be excluded. Here, we thus focused on a more detailed analysis of the 32 L. monocytogenes cluster 3 isolates from facility X (as well as one cluster 3 isolate from another nearby facility) to assess the short-term genomic evolution of L. monocytogenes in a food processing facility. We are not aware of any prior studies in which the rate of evolution and the time of the most common recent ancestor (MCRA) have been estimated for L. monocytogenes using a longitudinal set of >30 isolates collected over a period of >18 years from a single food processing facility. Inclusion of cluster 1 and 2 isolates provided a comparison group of isolates that were found in the same facility but did not show evidence of a long period of persistence. Phage acquisition, replacement, and loss, as well as identification of plasmids, were included in the genomic characterization, which was complemented with a phenotypic analysis of selected isolates representing different clusters. Specifically, reduced quaternary ammonium compound (QAC) sensitivity and the ability to attach to an abiotic surface, as well as growth and survival under different stress conditions, were assessed to probe for an association between these phenotypes and the presence of selected sanitizer tolerance genes, stress resistance genes, and a locus previously suggested to be associated with the ability to attach to surfaces.

## RESULTS

### Listeria monocytogenes from a cold-smoked salmon processing facility evolved slowly by point mutations and negative selection.

**FIG 2:**
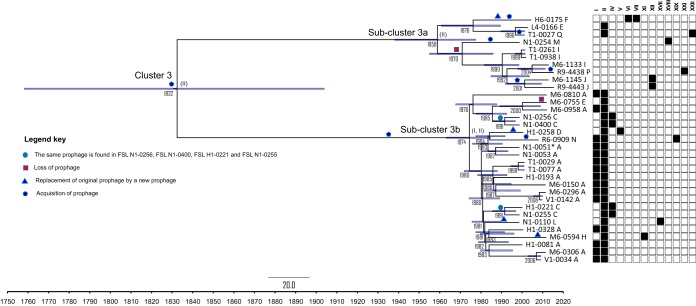
Tip-dated phylogeny of cluster 3 isolates. The phylogenetic tree was constructed using BEAST, version 2.5.2. Point estimates of the year when the common ancestor existed are shown under the associated nodes. Horizontal bars at the nodes represent the 95% HPD. Branch lengths are proportional to the estimated time since divergence. Events of acquisition (pentagon), loss (square), or replacement (triangle) of prophage sequences are indicated. The prophage profiles (PP) (Table 2) are shown next to the isolates’ identification numbers. Presence (filled squares) and absence (open squares) of prophage sequences (ϕ) are shown next to the phylogenetic tree. The prophage sequence (ϕ-XIX) that is shared by polyphyletic isolates is marked with a solid blue circle. Prophages ϕ-III, ϕ-VIII, ϕ-IX, ϕ-X, ϕ-XIII, ϕ-XIV, ϕ-XV, ϕ-XVI, and ϕ-XX were not identified among cluster 3 isolates. Prophages predicted to be present in the cluster 3 and subclusters 3a and 3b MRCA are shown in parenthesis. The cluster 3 isolate (FSL N1-0051) that was not isolated in facility X is indicated by an asterisk (*) after its name.

The 33 L. monocytogenes cluster 3 isolates obtained between 1998 and 2015 (32 from facility X and 1 from another facility) were analyzed using tip-dated phylogeny (Fig. 2). The mutation rate for a single-nucleotide change was estimated as 1.15 × 10^−7^ (95% highest posterior density [HPD] interval of 0.79 × 10^−7^ to 1.52 × 10^−7^) per nucleotide per year, which for L. monocytogenes is equivalent to 0.35 single-nucleotide changes per genome per year, or 1 change per genome every 2.9 years. The most recent common ancestor (MRCA) of all 33 cluster 3 isolates was estimated to have existed circa 1832 (95% HPD interval of 1759 to 1905), more than 100 years before the facility started operation. The MRCA for subcluster 3a was estimated to have existed circa 1958 (95% HPD interval of 1938 to 1978), within the time when the company started production in the facility. The MRCA for subcluster 3b was estimated to have existed circa 1974 (95% HPD interval of 1963 to 1985), which is around the time when the company expanded the facility (Fig. 2). One comparison isolate (FSL N1-0051) from another facility fell into subcluster 3b and differed from the most closely related facility X isolate (FSL N1-0053) by 11 SNPs (6).

The ratio of the number of nonsynonymous changes (Nc) per nonsynonymous site (Ns) over the number of synonymous changes (Sc) per synonymous site (Ss) (*dN*/*dS*, or ω) was <1.0 for cluster 3 as well as for subclusters 3a and 3b, suggesting that, in general, negative selection was the main force driving the evolution of these isolates, which appear to have persisted in facility X. More specifically, the 10 subcluster 3a isolates had 36 nonsynonymous and 26 synonymous changes (i.e., pairwise changes in coding regions identified among subcluster 3a isolates), which results in a ω of 0.41, while the 23 subcluster 3b isolates had 87 nonsynonymous and 31 synonymous changes, which results in a ω of 0.82. Between subclusters 3a and 3b, 49 nonsynonymous and 27 synonymous changes were identified, which results in a ω of 0.53. By comparison, the six cluster 1 isolates had 129 nonsynonymous and 61 synonymous changes, which results in a ω of 0.62. The two cluster 2 isolates had 4 nonsynonymous and 0 synonymous changes, which results in a ω of ∞.

### Among the SNPs that differentiate the isolates within the clusters, a number of mutations resulting in PMSCs could be identified.

Among the 78 SNPs that differentiate subcluster 3a and 3b isolates and that are located in protein-coding genes, two lead to premature stop codons (PMSCs). One of these two SNPs, found among all subcluster 3a isolates, is located in *lmo0524*, which encodes a sulfate transporter, while the other SNP resulting in a premature stop codon was found among all subcluster 3b isolates and is located in a gene encoding a putative homolog of the 2-succinyl-6-hydroxy-cyclohexadiene-1-carboxylate synthase (*lmo2074*). In addition, all 33 cluster 3 isolates also carry a premature stop codon in *inlA*, resulting in a 699-amino-acid (aa) protein, which cannot attach to the bacterial cell wall (7).

Among the 63 SNPs identified in protein-coding genes within subcluster 3a, 1 SNP resulting in a premature stop codon in two isolates was found in a gene encoding a protein of unknown function, DUF4866 (*lmo0451*), which is commonly found in human gut metagenomics studies (InterPro accession number IPR032357 [https://www.ebi.ac.uk/interpro/entry/IPR032357]) (Table 1). Among the 121 SNPs identified in protein-coding genes within subcluster 3b, 3 SNPs result in premature stop codons in genes encoding (i) an ATP-binding ABC transporter (protein encoded by *lmo1131*; two isolates), (ii) the iron-regulated surface determinant protein A (*lmo2185*; two isolates), and (iii) a thioredoxin-like protein (*lmo1903*; four isolates) putatively involved in cell redox homeostasis (Table 1). All three proteins with premature stop codons have signal peptide sequences, suggesting that they are all extracytoplasmic (Table 1).

**TABLE 1:** Single-nucleotide polymorphisms resulting in premature stop codons

Cluster and EGD-e homolog^*a*^	Protein	Protein length with PMSC(s) (aa)^*b*^	Full-length protein (aa)	Predicted location of full-length protein^*c*^	Isolate(s) carrying an allele with PMSC(s)
Cluster 1					
*inlA* (*lmo0433*)	Internalin A	491	800	Cell wall	FSL M6-0204, FSL R6-0665, FSL R6-0670, FSL R6-0682
*inlA* (*lmo0433*)	Internalin A	188	800	Cell wall	FSL H1-0506
*lmo0130*	Endonuclease	683	782	Cell wall	FSL H1-0506
*lmo0514*	Internalin-like protein	549	611	Cell wall	FSL M6-0204
*lmo2353*	Sodium, potassium, lithium, and rubidium/H^+^ antiporter	264	650	Membrane	FSL R6-0665, FSL R6-0670, FSL R6-0682
*lmo0634*	d-Tagatose-bisphosphate aldolase subunit	415	422	Cytoplasm	FSL N1-0013
*lmo2356*	Hypothetical protein	203	207	Unknown	FSL R6-0665, FSL R6-0670, FSL R6-0682, FSL N1-0013, FSL H1-0506
Cluster 3a					
*lmo0451*	Unknown function	88	251	Unknown	FSL M6-1133, FSL R9-4438
Cluster 3b					
*lmo1131*	ATP-binding ABC transporter	79	571	Cell membrane	FSL M6-0306, FSL V1-0034
*lmo2185*	Iron-regulated surface determinant protein A	4	569	Cell wall	FSL N1-0256, FSL N1-0400
*lmo1903*	Thioredoxin-like protein	78	157	Extracytoplasmic	FSL M6-0755, FSL M6-0958, FSL N1-0256, FSL N1-0400
Cluster 3					
*inlA* (*lmo0433*)	Internalin A	699	800	Cell wall	All isolates
Between subclusters 3a and 3b					
*lmo0524*	Sulfate transporter	95	553	Cell membrane	All sub-cluster 3a isolates
*lmo2074*	2-Succinyl-6-hydroxy-cyclohexadiene-1-carboxylate synthase	319	325	Cytoplasm	All sub-cluster 3b isolates

a Gene designation based on the EGD-e annotation described by Glaser et al. (93).

b PMSC, premature stop codon; aa, amino acid(s).

c Location predicted based on presence or absence of signal peptide, transmembrane domain, and/or cell wall-attaching domain (e.g., LPXTG).

Among the 240 SNPs identified in protein-coding genes among the six cluster 1 isolates, 7 SNPs resulted in premature stop codons in six genes. Among these SNPs, two independent SNPs lead to premature stop codons in *inlA*; one SNP is present in four isolates while the other SNP is present in a single isolate (Table 1). Only one cluster 1 isolate (FSL N1-0013) harbors a full-length *inlA* sequence. The other five SNPs resulting in premature stop codons were observed in genes encoding the following: (i) an endonuclease (protein encoded by *lmo0130*; one isolate); (ii) an internalin-like protein with a leucine-rich repeat (LRR) domain (*lmo0514*; one isolate)and with a premature stop codon interrupting translation of the protein before the cell wall-anchoring LPXTG motif, probably resulting in a secreted form of the protein; (iii) a sodium, potassium, lithium, and rubidium/H(+) antiporter (*lmo2353*; three isolates); (iv) a d-tagatose-bisphosphate aldolase subunit (*lmo0634*; one isolate); and (v) a hypothetical protein (*lmo2356;* 5 isolates) (Table 1). Given the location of the premature stop codons, the interrupted internalin A, the sodium, potassium, lithium, and rubidium/H(+) antiporter, and the internalin-like proteins are highly likely to show functional defects as both mutations in *inlA* and in the internalin-like-encoding genes result in proteins that will not be attached to the cell wall, while the predicted sodium, potassium, lithium, and rubidium/H(+) antiporter proteins have less than half of the amino acid length of the full-length protein (Table 1). No SNPs resulting in a premature stop codon were identified in *prfA*, which encodes the major L. monocytogenes transcriptional regulator of virulence genes.

### Short-term evolution of Listeria monocytogenes from a cold-smoked salmon processing facility is characterized by frequent prophage variation.

A total of 22 unique prophages (named here ϕ-I to ϕ-XXII) and 17 unique prophage profiles (PP; indicating a unique combination of prophages in a genome) were identified among the 42 L. monocytogenes isolates analyzed here (Table 2). The most common PP, PP-A, was observed among 14 subcluster 3b isolates. The second most common PP, PP-I (absence of any detectable prophages), was observed among three cluster 1 isolates and three subcluster 3a isolates; the third most common PP, PP-C, was observed among four subcluster 3b isolates. The fourth most common PP, PP-E, was observed among one cluster 1 isolate, one subcluster 3a isolate, and one subcluster 3b isolate. PP-B and PP-J were observed among the two cluster 2 isolates and two subcluster 3a isolates, respectively. The other 11 PPs were each unique to a single isolate (Table 2).

**TABLE 2:** Prophage profiles among the 42 isolates analyzed here

Prophage profile	No. of isolates	No. of prophages	Prophage(s)^*a*^	Cluster and/or subcluster(s)
PP-A	14	2	ϕ-I, ϕ-II	Subcluster 3b
PP-B	2	1	ϕ-III	Cluster 2
PP-C	4	2	ϕ-II, ϕ-IV	Subcluster 3b
PP-D	1	2	ϕ-II, ϕ-V	Subcluster 3b
PP-E	3	1	ϕ-II	Cluster 1, subcluster 3a, subcluster 3b
PP-F	1	2	ϕ-VI, ϕ-VII	Subcluster 3a
PP-G	1	3	ϕ-VIII, ϕ-IX, ϕ-X	Cluster 1
PP-H	1	2	ϕ-II, ϕ-XI	Subcluster 3b
PP-I	6	0^*b*^		Cluster 1 (*n* = 3), subcluster 3a (*n* = 3)
PP-J	2	1	ϕ-XII	Subcluster 3a
PP-K	1	4	ϕ-XIII, ϕ-XIV, ϕ-XV, ϕ-XVI	Cluster 1
PP-L	1	2	ϕ-II, ϕ-XVII	Subcluster 3b
PP-M	1	1	ϕ-XVIII	Subcluster 3a
PP-N	1	3	ϕ-I, ϕ-II, ϕ-XIX	Subcluster 3b
PP-O	1	1	ϕ-XX	FSL R9-4003 (unclustered)
PP-P	1	1	ϕ-XXI	Subcluster 3a
PP-Q	1	2	ϕ-II, ϕ-XXII	Subcluster 3a

a Prophages were identified using PHASTER. Only prophages classified as questionable or intact were considered. Prophage sequences with >95% coverage and >99% identity using blastn received the same identification.

b Isolates with no prophages identified were classified into profile PP-I.

Within cluster 3, events that appear to represent prophage acquisition (*n* = 7), prophage loss (*n* = 2), and prophage replacement (*n* = 6) were detected. Prophage replacement is defined here as the loss of a prophage that was present in the MRCA and acquisition of another prophage that was not present in the MRCA, with both events occurring within the same branch in the tip-dated phylogenetic tree (Fig. 2). The seven prophage acquisitions identified within cluster 3 occurred in subcluster 3a (*n* = 5) and in subcluster 3b (*n* = 2). The two prophage losses occurred in subcluster 3a (*n* = 1) and 3b (*n* = 1). The six prophage replacements identified in cluster 3 happened within subclusters 3a (*n* = 1) and 3b (*n* = 5) and therefore occurred after the MRCAs of these two subclusters, which were estimated as having existed circa 1958 (subcluster 3a) and 1974 (subcluster 3b). While four of the six replacement events involved single isolates, two replacement events involved multiple isolates. Specifically, isolates FSL H1-0221 and FSL N1-0255, which were isolated in 2000 and 1998, respectively, and had an MRCA that was estimated to have existed circa 1992, had the same PP (PP-C) as isolates FSL N1-0400 and FSL N1-0256, both of which were isolated in 1998 and also shared an MRCA estimated to have existed circa 1992 (Fig. 2). These two replacement events could be explained by two different hypotheses: (i) a horizontal transfer event between the isolates within subcluster 3b or (ii) two independent replacement events through the acquisition of the same prophage sequence in these isolates. Although these four isolates belong to subcluster 3b, they are polyphyletic within the cluster. In addition, a cluster 1 isolate (FSL H1-0506) harbored the same prophage (ϕ-II) found otherwise only among subcluster 3b isolates, also suggesting either horizontal transfer of the prophage between the cluster 1 isolate and a subcluster 3b isolate or coacquisition of similar prophages by subcluster 3b and the cluster 1 isolate; the ϕ-II sequence from FSL H1-0506 and the subcluster 3b ϕ-II sequence showed no indels and only 47 mismatches in 53,106 nucleotides (nt).

### Stress resistance genes present in the chromosome and plasmids were associated with distinct clusters, subclusters, and single isolates.

All isolates within clusters 1 and 2 and subclusters 3a and 3b were identified as carrying plasmids that were specific to each cluster or subcluster, suggesting limited horizontal transfer of plasmids within the facility between clusters and subclusters (Table 3). A phylogenetic analysis using the extracted nucleotide sequence of the plasmid replication protein (primase protein) showed that the six plasmids identified among the isolates correspond to three phylogenetically distinct groups (A, B, and C) (see Fig. S1 in the supplemental material). The subcluster 3a plasmid (pLM-3a) represented group C, while the subcluster 3b plasmids (pLM-3b-A and pLM-3b-B) represented groups A and B. While the plasmid found in the unclustered isolate FSL R9-4003 (pLM-R94003) also represented group A, it was distinct from plasmid pLM-3b-A; similarly, while the plasmid found in both cluster 2 isolates (pLM-2) also represented group B, this plasmid is different from pLM-3b-B. The plasmid found in the six cluster 1 isolates (pLM-1) also represented group C; this plasmid is similar to group C plasmid pLM-3a, as detailed below.

**TABLE 3:** Plasmids identified

Cluster or subcluster	Size of plasmid (kb)^*a*^	No. of predicted protein-coding genes	Plasmid replication protein group^*b*^
1	62	61	C
2	77	81	B
3a	67	68	C
3b	81	88	B
	58	61	A
FSL R9-4003 (unclustered)	71	77	A

a Size of the plasmids was determined as the sum of contigs matching the stand-alone BLAST plasmid database.

b Groups were defined based on a phylogenetic analysis (see Fig. S1 in the supplemental material) using the extracted nucleotide sequence of the plasmid replication protein (primase protein).

pLM-1 is 62 kb long and harbors 61 genes, including genes encoding proteins putatively involved in heat resistance (*clpL*) and reduced sensitivity to cadmium (*cadA* and *cadC*) (Table 4). pLM-2 is 77 kb long and harbors 81 genes, including genes encoding proteins putatively involved in cation transport (*zosA*), osmotic stress (*gbuC*), oxidative stress (*npr*), and reduced sensitivity to copper (*copY*), cadmium (*cadA* and *cadC*), and QAC (*bcrABC*) (Table 4). pLM-3a is 67 kb long and carries 68 genes, including all the genes expressed by pLM-1 with the addition of genes encoding a DNA invertase, two putative transposases, and three hypothetical proteins, as well as the *bcrABC* operon, which confers reduced sensitivity to QAC. In addition to the presence of these additional seven genes in pLM-3a, only five mismatches and one indel differentiated pLM-3a from pLM-1, suggesting a common origin of these two plasmids, with insertion of a transposon into pLM-1 likely leading to the emergence of pLM-3a. Subcluster 3b isolates were the only isolates to carry two plasmids. pLM-3b-A is 58 kb long and harbors 61 genes, including genes encoding proteins putatively involved in heat resistance (*clpL*), osmotic stress (*gbuC*), oxidative stress (*npr*), cation transport (*zosA*), and reduced sensitivity to copper (*mco* and *copB*) (Table 4). pLM-3b-B is 81 kb long and expresses 88 genes, including 6 genes encoding proteins putatively involved in reduced sensitivity to copper (*copY*), cadmium (*cadA* and *cadC*), and QAC (*bcrABC*) (Table 4). Interestingly, the two plasmids present in all subcluster 3b isolates carried most of the genes present in the plasmids found in the other isolates (Table 4), with several additional stress response genes present in the subcluster 3b plasmids. pLM-R94003 is 71 kb long and expresses 77 genes, including genes encoding proteins putatively involved in osmotic stress (*gbuC*), heat resistance (*clpL*), oxidative stress (*npr*), cation transport (*zosA*), and reduced sensitivity to copper (*mco* and *copB*), cadmium (*cadA* and *cadC*), mercury (*merR* and *merAB*), and QAC (*bcrABC*) (Table 4).

**TABLE 4:** Stress response accessory genes found among the three clusters

Location and type of genetic material	Cluster, subcluster, or isolate^*a*^					Predicted resistance function(s)	Reference(s)
	1	2	3a	3b	FSL R9-4003		
Plasmid-borne genes and operons							
*bcrABC*	−	+	+	+	+	QAC	94
*clpL*	+	−	+	+	+	Heat	41
*gbuC*	−	+	−	+	+	Osmotic stress	42, 43
*npr*	−	+	−	+	+	Oxidative stress	44, 45
*cadA*	+	+	+	+	+	Heavy metal	47
*cadC*	+	+	+	+	+	Heavy metal	47
*copB*	−	−	−	+	+	Heavy metal	49, 50
*copY*	−	+	−	+	−	Heavy metal	51
*mco*	−	−	−	+	+	Heavy metal	48, 50
*merAB*	−	−	−	−	+	Heavy metal	53
*merR*	−	−	−	−	+	Heavy metal	53
*zosA*	−	+	−	+	+	Heavy metal	52
Chromosome-borne gene and islets							
*qacH*	FSL M6-0204	−	−	−	−	QAC	10
SSI-1	−	−	+	+	+	Acidic stress and high salt concentrations	8
SSI-2	+	−	−	−	−	Alkaline and oxidative stress	9

a Plus and minus signs indicate the presence and absence, respectively, of the element.

In addition to the stress resistance genes found in the plasmids, a number of other stress resistance genes were found in the chromosome of the isolates. The survival stress islet 1 (SSI-1), which has been suggested to confer a growth advantage under acidic stress and high salt concentrations (8), was found among all 33 cluster 3 isolates and in the unclustered isolate FSL R9-4003 but was absent from all cluster 1 and cluster 2 isolates (Table 4). The survival stress islet 2 (SSI-2), which has been shown to confer a survival advantage under alkaline and oxidative stress (9), was found among all cluster 1 isolates but was absent from all other isolates (Table 4). Therefore, cluster 2 isolates lacked both SSI-1 and SSI-2. In addition, the presence of the stress survival islets was significantly associated with the cluster 3 isolates from facility X (Fisher’s exact test, *P* ≤ 0.05). Although isolates carrying pLM-1 lacked the QAC tolerance operon *bcrABC*, one cluster 1 isolate (FSL M6-0204) was found to harbor the Tn*6188* transposon (Table 4), which includes the QAC tolerance gene *qacH* (10).

### Resistance genes *qacH* and *bcrABC* confer reduced sensitivity to QAC sanitizers and are associated with persistent isolates.

In order to assess whether the presence of *bcrABC* or *qacH* could confer reduced sensitivity to QAC sanitizers, five isolates representing the three observed genotypes (i.e., presence of *qacH* and absence of *bcrABC*, presence of *bcrABC* and absence of *qacH*, and absence of *bcrABC* and *qacH*) and four clusters/subclusters (i.e., clusters 1 and 2 and subclusters 3a and 3b) were incubated in the presence of four QAC sanitizers: benzalkonium chloride (BC), cetylpyridinium chloride (CPC), benzethonium chloride (BZT), and benzyl-C_12_-C_16_-alkyl-dimethyl-ammonium chloride (Weiquat) (Table 5 and Fig. S2). All isolates carrying either *qacH* or *bcrABC* showed reduced sensitivity to all four QAC sanitizers, as indicated by MIC values higher than those for the isolate lacking the sanitizer resistance genes (Table 5 and Fig. S2).

**TABLE 5:** MICs of selected QAC sanitizers

Strain	Cluster or subcluster	Gene(s)	MIC (mg/liter) of^*a*^:			Weiquat MIC (%)
			BC	BZT	CPC	
FSL H1-0506	1	None	1	2	1	0.001
FSL M6-0204	1	*qacH*	3	4	3^*d*^	0.004^*e*^
FSL H1-0322	2	*bcrABC*	4^*b*^	7^*c*^	3^*d*^	0.004
FSL T1-0027	3a	*bcrABC*	3	5	2	0.004
FSL T1-0077	3b	*bcrABC*	3	6	2	0.004

a The MIC is defined as the value at which no growth was detected (at the detection threshold of an OD_600_ of 0.15) for any of the three biological replicates after 24 h of incubation. BC, benzalkonium chloride; BZT, benzethonium chloride; CPC, cetylpyridinium chloride.

b One of three biological replicates passed the detection threshold at 3 mg/liter BC.

c One of three biological replicates passed the detection threshold at 6 mg/liter BZT.

d One of three biological replicates passed the detection threshold at 2 mg/liter CPC.

e One of three biological replicates passed the detection threshold at 0.003% Weiquat.

The three isolates carrying *bcrABC* showed equal or higher MIC values than the isolates carrying *qacH* when exposed to low concentrations of BC, BZT, and Weiquat (Table 5). Interestingly, not all *bcrABC*-carrying isolates had the same MIC values; the cluster 2 isolate FSL H1-0322 showed higher MIC values for all four QAC sanitizers than both cluster 3 isolates (FSL T1-0027 and FSL T1-0077). The reduced sensitivity observed among the isolates carrying either *qacH* or *bcrABC* was confirmed in a follow-up assessment of growth of seven additional isolates (FSL R6-0670, FSL H1-0159, FSL R9-4443, FSL L4-0166, FSL T1-0938, FSL N1-0053, and FSL V1-0034) in the presence of BC. Confirming the previous results, isolates lacking the tolerance genes (FSL H1-0506 and FSL R6-0670) had a 2-ppm-lower MIC than the isolates carrying either tolerance locus (Fig. S3).

The presence of QAC sanitizer tolerance genes was also assessed for association with cluster 3 isolates from facility X. The *bcrABC* operon was identified in all isolates belonging to cluster 3, as well as in two transient isolates belonging to cluster 2. Only one isolate of the five transient isolates belonging to cluster 1 carried *qacH*. Therefore, 32 of the 32 cluster 3 isolates, which were putatively persistent in facility X, harbored a QAC tolerance gene, while 3 of 7 cluster 1 and cluster 2 isolates, which were putatively transient in facility X, harbored a QAC gene (Fisher’s exact test, *P* ≤ 0.0005).

### Isolates representing the different clusters and subclusters differed in their abilities to grow under various stress conditions, but those differences were not related to the presence or absence of identified stress resistance genes.

Growth of the five isolates that were assessed for reduced sanitizer sensitivity was also evaluated under an optimal condition (i.e., 37°C) and under different stress conditions, including in brain heart infusion (BHI) broth at (i) pH 5.5, (ii) 15.5°C, (iii) 40°C, (iv) water activity (a_w_) of 0.95, and (v) 6.5% NaCl. Growth parameters assessed included (i) growth rate and (ii) the optical density at 600 nm (OD_600_) at early stationary phase (OD_ESP_) under these conditions. All isolates showed the highest growth rate at 37°C. Significant differences in growth rates across isolates were detected under all conditions except for growth at 15.5°C (Fig. S4A). The range of growth rates was largest for growth at 40°C (0.9 to 1.17 h^−1^) and pH 5.5 (0.66 to 0.91 h^−1^). For both of these conditions, as well as for growth in the presence of 6.5% NaCl, the two cluster 3 isolates showed significant differences in growth rates; under all three of these conditions, the subcluster 3b isolate showed a lower growth rate. For example, at pH 5.5, the subcluster 3b isolate showed a growth rate of 0.66 h^−1^ whereas that of the subcluster 3a isolate was 0.91 h^−1^. Interestingly, the two cluster 1 isolates tested also showed significant differences when grown at 40°C and under high-salt conditions. For example, at 40°C, isolate FSL H1-0506 showed a growth rate of 1.12 h^−1^, and isolate FSL M6-0204 had a growth rate of 1.04 h^−1^.

The highest OD_ESP_, indicating the highest bacterial density, across all conditions was reached when isolates were grown at 37°C, and the lowest OD_ESP_ was reached when isolates were grown under high-salt conditions (Fig. S4B). Significant differences in OD_ESP_ values were identified across isolates for all conditions tested. The range for OD_ESP_ values was largest when isolates were grown under high-salt conditions, with OD_ESP_ values ranging from an OD_600_ of 0.31 to an OD_600_ of 0.52. Both cluster 3 isolates showed significant differences in OD_ESP_ values under all conditions, with the subcluster 3b isolate showing a consistently lower OD_ESP_ than the subcluster 3a isolate, consistent with the reduced growth rate observed for the subcluster 3b isolate under some conditions. In contrast, both cluster 1 isolates reached OD_ESP_ values that did not differ significantly when grown under conditions of 40°C, reduced water activity, and pH 5.5 and reached significantly higher OD_ESP_ values than all other isolates when grown under conditions of 40°C and pH 5.5 (Fig. S4B).

### Isolates representing the clusters and subclusters showed no significant difference in sensitivities to oxidative stress.

Isolates were exposed to 10 mM cumene hydrogen peroxide (CUHP) for 2 h to assess their survival under oxidative stress. The average log reduction of all isolates was not significantly different from each other, as indicated by the results of the analysis of variance (ANOVA) (*P* = 0.10) (Fig. S5).

### Cluster 2 isolates showed lower attachment levels than isolates representing cluster 1 or 3.

**FIG 3:**
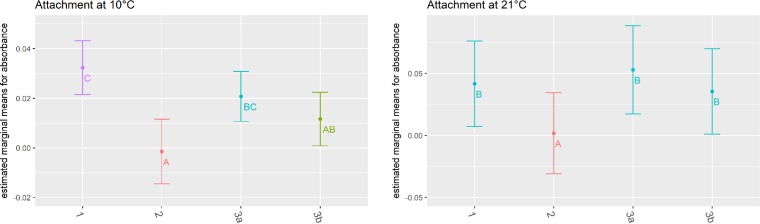
Attachment by cluster. Estimated marginal means of absorbance measured at an OD_600_ by cluster (*x* axis) at 10°C and 21°C. Colors match the letter codes that are based on *post hoc* Tukey analysis; data points that do not share the same letter are significantly different. The bars indicate the estimated upper and lower 95% confidence intervals based on the linear mixed regression model with three biological replicates.

Twelve isolates, as well as a Pseudomonas aeruginosa control strain, were incubated in 96-well microtiter plates for 120 h at 10°C and 21°C to assess their attachment capabilities as measured by absorbance of a crystal violet stain. Overall, a significant difference in attachment levels was observed across the 12 isolates (ANOVA, *P* < 0.001). Overall, higher absorbance (indicating more attached cells) was obtained when isolates were incubated at 21°C than at 10°C (ANOVA, *P* < 0.001) (Table S3 and Fig. S6). The high initial inoculum (i.e., ∼10^7^ CFU/ml) combined with the long incubation time (i.e., 120 h) before measurements were taken suggests that cells had reached stationary phase when measurements were taken at both 10°C and 21°C. However, we cannot completely rule out the possibility that the differences observed between the two temperatures are due to the fact that a higher cell density was reached at 21°C than at 10°C. A significant difference in attachment levels was also observed for clusters and subclusters (ANOVA, *P* < 0.001) (Table S4). Under incubation at 10°C, the cluster 2 isolates showed the lowest attachment while the cluster 1 isolate (FSL M6-0204) showed the highest attachment. Similarly, when isolates were incubated at 21°C, cluster 2 isolates showed the lowest attachment of all isolates. At 21°C, no significant difference was observed for the attachment of cluster 1 and subclusters 3a and 3b (Fig. 3). Transient isolates (cluster 1 and 2) showed significantly lower attachment than persistent isolates (cluster 3) when incubated at 21°C (ANOVA, *P* = 0.037) (Table S5). It has been suggested that prophage integration into the gene *comK* is positively associated with the ability of L. monocytogenes to attach to surfaces (11). Here, the attachment level of the isolates measured by their absorbance of a crystal violet stain was not associated with the presence or absence of a prophage in *comK* (ANOVA, *P* = 0.788) (Table S6).

## DISCUSSION

L. monocytogenes has previously been reported to survive and persist in food processing environments for many years (2, 3), facilitating repeat contamination of the finished product. Studies assessing the genetic evolution of L. monocytogenes occurring in a single food facility over several years are generally lacking. This information is critical to interpret WGS data as the technology is being increasingly adopted by the industry for the root cause analysis of a pathogen contamination event. Thus, in this study, we applied WGS data to analyze a set of 40 L. monocytogenes isolates collected from 1998 to 2015 from a cold-smoked salmon processing facility to provide an in-depth characterization of the evolution of this foodborne pathogen in a food-associated environment. Our results indicated the following: (i) a lower mutation rate among environmental persistent isolates than clinical isolates, (ii) the possibility of multiple reintroductions with closely related isolates (cluster 1, ST121 isolates), (iii) long-term persistence in a food processing environment (i.e., subcluster 3b isolates, which were obtained between 1998 and 2012), and (iv) the possibility of strain diversification within the facility (i.e., diversification of cluster 3 into subclusters 3a and 3b). These results can help regulatory agencies and the food industry to better interpret WGS data used in traceback investigations and root cause analyses, especially in smoked-fish processing facilities or other facilities that share similar environmental characteristics (e.g., low temperature and high moisture). For example, our results suggest that traceback investigations should not take into consideration prophage sequences as these sequences evolve much faster through gain, loss, replacement, and, potentially recombination, than the chromosome backbone sequence.

### L. monocytogenes isolates obtained over time in a smoked-seafood facility show an overall low rate of point mutations per year.

Excluding prophage sequences, the evolution of the isolates within the cold-smoked salmon processing facility was characterized by a low rate (per year) of point mutations leading to observable single-nucleotide polymorphisms (SNPs). Our estimated rate of 1.15 × 10^−7^ substitutions per site per year is lower than the estimated rate (2.4 × 10^−7^ substitutions per site per year) obtained from 12 L. monocytogenes lineage II clinical isolates from humans and animals (12). Although the 2-fold-lower rate obtained in this study could be a result of slightly different approaches used to obtain the estimates or of the different number of isolates used in each study, we hypothesize that isolates from the cold-smoked processing environment are evolving more slowly than clinical isolates. The cold-smoked processing environment is kept at low temperatures, typically lower than 10°C, which, although allowing for growth of L. monocytogenes, significantly increases the generation time of the organism compared to that with the optimal growth temperature (typically 30 to 37°C). In addition to differences in generation time and variation in population sizes, the direction (i.e., negative or positive) and strength of natural selection could also contribute to differences in the rate of changes per site per year (13). Moreover, 99% of human infections are considered to be foodborne, suggesting that human clinical isolates also evolved, at least partially, in food processing environments. Therefore, the differences between the mutation rate estimated here and the mutation rate previously estimated based on mainly human clinical isolates may also reflect a difference related to growth in environments associated with different commodities (e.g., ready-to-eat delicatessen foods, cheese, and cold-smoked salmon).

The isolates in the three clusters analyzed here (1, 3a, and 3b) evolved, on average, by negative, close to neutral, selection, as evidenced by ω (*dN*/*dS*) values lower than but close to 1. However, at least some gene-specific positive selection toward loss of function can be hypothesized, for example, for *inlA*. Within the 42 isolates analyzed, four different changes leading to premature stop codons (PMSCs) in *inlA* were identified. Moreover, within cluster 1, two distinct *inlA* PMSCs were detected, suggesting a strong selective pressure for loss of function of the internalin A protein. Interestingly, only three isolates within our data set presented full-length *inlA* sequences: the two cluster 2 isolates (FSL H1-0159 and FSL H1-0322) and one cluster 1 isolate (FSL N1-0013), which was isolated from a food product from a different facility. L. monocytogenes isolates carrying PMSCs in *inlA* have been widely isolated from foods and food processing and food retail environments (14–18). However, these PMSC genotypes are rarely found among isolates collected from nonfood environments, animals, and humans (14, 16, 19, 20). L. monocytogenes isolates carrying an *inlA* PMSC had previously been shown to have reduced ability to invade intestinal epithelial cells and reduced virulence in animal models (21, 22), suggesting that 39 of the 42 isolates analyzed here may be virulence attenuated. Although the selective pressure behind the internalin A loss of function has not been revealed, our data suggest that at least some food-associated environments, such as the cold-smoked salmon facility sampled in this study, present conditions that favor isolates with a truncated internalin A protein.

Evolution by positive selection has also been previously observed in L. monocytogenes genes that show multiple premature stop codons, such as *inlA* and *flaR* (2, 21), suggesting a selective pressure for amino acid changes or nonexpression of the full-length proteins encoded by these genes in certain environments or under certain conditions. In addition to internalin A, isolates characterized here presented at least seven other genes that encode proteins that are located outside the cytoplasm (i.e., membrane attached, cell wall attached, or secreted) and that had premature stop codons that reduced the length of the protein to less than 90% of the full length (i.e., the length without the premature stop codon). A previous genome-wide study of positive selection in L. monocytogenes also showed that genes encoding proteins involved in cell wall and membrane biogenesis were significantly more likely to have evolved by positive selection, and several genes encoding proteins that are attached to the cell membrane or the cell wall have previously been reported to evolve by positive selection (2). While these findings may suggest that selected genes encoding surface molecules may be under positive selection for loss of function during survival in selected non-host-associated environments (e.g., processing plants), further work will be needed to test this hypothesis.

### Isolates obtained from a smoked-seafood facility over time show fast diversifying and convergent evolution of prophage sequences.

Despite the low single-nucleotide mutation rate observed in the genomes of the isolates, fast diversification of prophage sequences was detected. Fast prophage diversification in food processing settings has been previously described (2, 18, 23) although one study reported limited diversification within the prophage inserted into *comK* for isolates collected from 15 food facilities (11). Fast prophage diversification has also been shown among human isolates (23–25) and could result in closely related isolates being assigned to different subtypes, depending on the molecular subtyping method used (e.g., pulsed-field gel electrophoresis [PFGE]) (25, 26). It is important to note, however, that prophage acquisitions and losses do not follow a vertical evolution, like that of SNPs; therefore, prophage profiles should not be used for genetic clustering of isolates, and analysis of prophage presence/absence (or prophage-associated SNPs) cannot be used to establish or further refine the time of a most recent common ancestor.

### Reduced sensitivity to QAC sanitizer was associated with the presence of QAC tolerance genes.

Out of the 42 isolates in our data set, 37 harbored genes with predicted functions relevant to reduced QAC sensitivity. Thirty-six isolates, representing all cluster 2 and cluster 3 isolates as well as FSL R9-4003 (unclustered isolate) harbored the plasmid-borne operon *bcrABC*, and one isolate, FSL M6-0204 (cluster 1), harbored the transposon-associated gene *qacH*. The remaining five cluster 1 isolates harbored no QAC tolerance genes. Both genes, *bcrABC* and *qacH*, encode efflux pumps belonging to the small multidrug resistance family (SMR) and have been previously shown to confer reduced sensitivity to low levels of several QAC sanitizers (10, 27, 28). QAC sanitizers are often used in food processing facilities and have also been applied in facility X, which was the source of the 40 isolates studied here. The cationic, cell membrane-acting sanitizer is usually applied at concentrations of 200 to 400 ppm and has been shown to effectively reduce L. monocytogenes (more than a 3-log reduction) (29–32). However, traces of highly diluted sanitizer can reach niches harboring L. monocytogenes, and isolates carrying the QAC tolerance genes might have a growth advantage at low-level sanitizer concentrations (32). Isolates from this study, carrying either *bcrABC* or *qacH*, exhibited 3- to 4-fold-reduced sensitivity toward low concentrations of QAC sanitizers compared to that of the isolate lacking these tolerance genes. This is consistent with a previous study in which a *qacH* deletion mutant was 3-fold more sensitive than the wild type when grown on medium with BC (10). Similarly, a study by Møretrø et al. found that isolates carrying the *bcrABC* operon had 2.5- to 5-fold-higher MICs when grown in medium with BC than isolates lacking *bcrABC* and *qacH* (27). In this study, as well as in previous studies, isolates with QAC tolerance genes have been shown to be associated with repeated isolation in a food processing environment and were shown to confer a growth advantage when exposed to low concentrations of QAC sanitizers (33–36). For example, a previous study found that isolates that shared the same sequence type and carried a plasmid expressing the *bcrABC* operon became the dominant sequence type in the second and third year of sampling in a food processing environment (36).

### Presence of stress resistance genes is correlated with phylogenetic clusters but was not associated with growth advantages in a phenotypic characterization.

While our study showed that the previously reported stress survival islets 1 and 2 (SSI-1 and -2, respectively) were associated with clusters and subclusters, we were not able to identify clear growth or stress response phenotypes associated with the presence of these islets. SSI-1 was detected among all cluster 3 isolates; this islet has been suggested to contribute to growth under low pH (pH 4.8), high salt (7.5% NaCl), and a combination of both (pH 5.2 and 5% NaCl) (8) as well as to biofilm formation at 30°C (37) and salt-induced nisin resistance (38). While 34 of 42 isolates in this study harbored SSI-1, only 3 of 12 isolates from a cold-smoked processing facility in Ireland harbored this islet (39). All cluster 1 isolates (ST121; CC121) harbored SSI-2, which has been suggested to confer resistance to alkaline and oxidative stress (9). SSI-2 was also shown to be present in all 77 ST121 isolates screened in two previous studies (23, 40), suggesting that this islet is widespread among this sequence type. Interestingly, the two cluster 2 isolates did not harbor either SSI-1 or SSI-2. These two isolates were isolated only in March and June of 2000 while isolates carrying either SSI-1 or SSI-2 were isolated from 1998 through 2015, suggesting that the presence of SSI-1 or SSI-2 may be associated with persistence, at least among the isolates characterized here.

In addition to the two islets found in the chromosome of some isolates, several other stress resistance genes were detected in the plasmids identified here. For example, the subcluster 3b plasmid pLM-3b-A includes (i) *clpL*, which has been reported to enhance heat tolerance (41), and (ii) other genes that have annotated functions that suggest they confer reduced sensitivity to osmotic stress (*gbuC*) (42, 43) and oxidative stress (*npr*) (44, 45).

Isolates were selected to represent each genotype identified in this study for phenotypic analyses by assessing their growth under different environmental conditions. While the number of isolates included in the initial phenotypic analyses might represent a limitation to the study, given the few SNP differences observed between isolates within the same cluster or subcluster, a single isolate representing each genotype was considered sufficient for the scope of this study. When isolates were phenotypically characterized, a clear growth advantage (e.g., higher growth rate or higher optical density in stationary phase) was not associated with the presence of a given plasmid or stress resistance islet (i.e., SSI-1 or SSI-2). For example, the subcluster 3b isolate FSL T1-0077 grew more slowly and to a lower density (OD_ESP_) than other isolates (including the subcluster 3a isolate) when grown under a high salt concentration (6.5% NaCl) even though this isolate carried two osmotic stress resistance loci (*gbuC* and SSI-1), while all other isolates carried one or none of these genes. Similarly, while SSI-1 has previously been reported to provide enhanced acid resistance, one of the isolates with SSI-1 (i.e., the subcluster 3b) showed the lowest growth rate at pH 5.5 even though the other isolate with SSI-1 (i.e., the subcluster 3a) showed the highest growth rate under these conditions; this suggests that multiple factors, not just the presence of SSI-1, affect acid resistance, at least at the pH tested. When the growth rate at 40°C was assessed, a growth advantage for isolates carrying *clpL* was not observed. This finding is in agreement with previous growth studies, which showed that an L. monocytogenes 10403S strain with a chromosomally integrated *clpL* did not show any growth advantage at 42°C compared to growth of the wild-type strain (41). Furthermore, we did not find any evidence for significant differences in oxidative stress survival rates among the tested isolates even though previous phenotypic studies (8, 9, 41) with isogenic mutants indicated that the presence of SSI-2 (which was found only in some of the isolates tested here) provided increased oxidative stress resistance. Consistent with the growth analysis under various stresses, it is possible that other genes play a larger role in the overall stress response than a single resistance gene or stress survival islet. Overall, our data suggest that while the presence of specific stress islets and stress genes may be associated with enhanced stress resistance in experiments with isogenic mutants, fitness advantages associated with the presence of these genes in wild-type isolates may be more difficult to assess. Interestingly, we also found an indication for a lower growth rate of the subcluster 3b isolate under most conditions tested, which could be related to the fact that the subcluster 3b isolate (as well as all other subcluster 3b isolates) carries two plasmids, in contrast to the cluster 1 and 2 and subcluster 3a isolates that each had only one plasmid. Carrying multiple plasmids can constitute a metabolic burden and can reduce the overall growth rate (46). The possible interactions between putative advantages associated with the presence of selected stress response genes and metabolic burdens associated with the presence of additional genes (particularly on plasmids) likely further complicate meaningful phenotypic assessment of wild-type isolates for food processing-associated environments.

In addition to the environmental stress genes, six heavy metal resistance genes shown to provide resistance to cadmium (*cadA* and *cadC*) (47) or with predicted functions suggesting involvement in resistance to high levels of copper (*mco*, *copB*, and *copY*) (48–51) and an ATPase transporter with affinity to an undefined cation (*zosA*) (52) were also detected in the two subcluster 3b plasmids. In addition, the plasmid found in cluster 1 and subcluster 3a also harbored *cadA* and *cadC*, while the cluster 2 plasmid harbored *copY*, *zosA*, *cadA*, and *cadC*. Interestingly, FSL R9-4003, the only isolate to not cluster with any other isolate in our data set harbored one plasmid that expressed three genes (*merA*, *merB*, and *merR*) with functions that had previously been associated with mercury (Hg^II+^) detoxication (53, 54). Sequence searches against the NCBI nonredundant (nr) database showed no matches for the sequences to any L. monocytogenes sequence available in the database, so we are not aware of these mercury detoxication genes being found previously in L. monocytogenes. Overall, the broad presence of genes with putative roles in heavy metal resistance is consistent with a number of previous reports that indicated the frequent presence of cadmium and arsenic resistance in *Listeria* (55–59) and raises interesting questions on possible roles of these genes in L. monocytogenes persistence, such as possible cross-protection against non-heavy metal-containing biocides.

### Attachment of isolates at room temperature (21°C) is associated with persistence.

Our data suggest that persistent isolates can attach better than transient isolates at 21°C but not at 10°C, which corresponds to the facility’s temperature. Some previous studies have linked the potential of certain L. monocytogenes strains to become persistent in food processing facilities to their increased ability to attach to abiotic surfaces (60, 61), among other characteristics that allow those strains to survive and grow in food processing environments. However, other previous studies have suggested that persistent and transient strains do not differ in their attachment capabilities (34, 62, 63); for example, Cherifi et al. reported that isolates classified as persistent and transient based on PFGE subtyping did not show significant differences in attachment, using the same assay used here (34). Interestingly, a study by Verghese et al. had previously suggested that L. monocytogenes isolates with prophage integration in *comK* showed higher densities of attached cells than isolates with an intact *comK* sequence (i.e., no prophage integrated into *comK*) (11). The isolate set evaluated here did not reflect these findings as prophage integration in *comK* was not associated with attachment in our study. These data further support the idea that it is unlikely that a single genetic feature facilitates increased attachment of L. monocytogenes, consistent with other previous studies (reviewed in reference 3). Studies have shown that flagella play a key role in L. monocytogenes attachment to surfaces (64, 65) and that the expression of flagellin is dependent on temperature as it is expressed at 30°C or below (66, 67). The temperatures chosen here, including for pregrowth, as well as the incubation temperature during the attachment assay provided equal conditions for flagellum formation for all strains. Our data also indicate that attachment is higher when isolates are incubated at 21°C than at 10°C, possibly at least partially as a result of a final higher cell density at 21°C than at 10°C. The same observation was made in previous studies that compared attachment of L. monocytogenes strains at these temperatures (62, 68). While future studies may provide new insights into strain characteristics that may enhance the ability of L. monocytogenes to establish persistence, the overall body of literature still seems to support the idea that a large number of L. monocytogenes strains can survive in processing facilities over time, given appropriate environmental conditions (e.g., niches), nutrient availability, temperature, and possibly the presence of other bacteria that may allow for mixed-biofilm establishment (62, 69–71).

### Conclusions.

In this study, we showed that under environmental conditions found in a fish-smoking facility, L. monocytogenes evolves more slowly than previously estimated based on human and animal isolates. In addition, we have also shown that prophage diversification is widespread and occurs much faster than single-nucleotide diversification. Hence, isolation of L. monocytogenes strains with few SNP differences in different locations (e.g., supplier plants and receiving plants) is possible, highlighting the importance of epidemiological and detailed isolate metadata for interpreting WGS data in traceback investigations. For example, while isolation of nearly identical isolates from food contact equipment immediately after sanitation would be indicative of persistence, repeat isolation in a high-traffic raw material area during production could also be due to reintroduction. This challenge is supported by the fact that we identified an isolate closely related to subcluster 3b, which persisted in the facility X evaluated here, in another nearby facility, similar to findings of a previous study which also identified closely related L. monocytogenes isolates (<4 SNP differences) in retail facilities in two different states in the United States (72). While we believe that this study provides key results that can help regulatory agencies and the food industry to better interpret L. monocytogenes WGS data from food and food-associated isolates, future similar studies in facilities with different environmental conditions will be needed to provide a broader context and more generalizable findings. Importantly, our data also suggest that MRCA estimates may be able to help identify specific events (e.g., expansions) that may have been associated with the introduction of persistent L. monocytogenes; this approach may be valuable for root cause analysis efforts.

## MATERIALS AND METHODS

### Isolates and WGS data.

The 42 isolates used in this study have previously been described (6) and included (i) 40 isolates previously classified into ribotype DUP-1062 and isolated from a single cold-smoked salmon processing facility and (ii) two additional comparison isolates (FSL N1-0013 and FSL N1-0051, representing cluster 1 and subcluster 3b, respectively), which were also previously classified into ribotype DUP-1062 but were isolated from two other cold-smoked salmon processing facilities, geographically close to the facility where the 40 isolates were obtained. WGS data for all 42 isolates, including (i) *de novo* assembly and quality assessment of the assemblies, (ii) identification of high-quality single-nucleotide polymorphisms (hqSNPs) among cluster 1 and 2 and subcluster 3a and 3b isolates, and (iii) classification of all isolates based on seven-gene multilocus sequence typing (ST), have also been previously reported (6). In this study, Prokka, version 1.12, was used to annotate the 42 L. monocytogenes genomes using default parameters for Gram-positive bacteria (73).

### Reference-free single-nucleotide polymorphism analysis.

The kSNP3 program was used to identify core SNPs among the 42 genomes included in this study (74) and 17 closed genomes downloaded from the NCBI RefSeq database, which represent a subset of the genomes used to create the kSNP3 tree previously reported by Jagadeesan et al. (6). These 17 genomes represented 16 isolates of lineage II, which is the same lineage as the 42 isolates used in this study, and one lineage I strain (F2365), which was used as an outgroup. The 16 lineage II isolates were selected (i) to represent the genetic diversity within lineage II and (ii) to include genomes that had previously been shown to cluster within or between clusters 1, 2, and 3 (6). A maximum likelihood tree was constructed based on the core SNP matrix using RAxML (version 8.2.12) and the GTRCAT model (75). Clustering confidence was obtained by 1,000 bootstraps. The tree was rooted using the outgroup strain (F2365).

### High-quality single-nucleotide polymorphism analysis.

While separate hqSNP analyses for subclusters 3a and 3b isolates were previously reported (6), identification of hqSNPs among all cluster 3 isolates was newly performed here. The FDA Center for Food Safety and Applied Nutrition (CFSAN) SNP pipeline, version 1.0.0. (76), was used with default parameters and with the high-quality draft assembly of FSL T1-0027 (subcluster 3a) as a reference for read mapping. Using a default threshold of no more than three SNPs within a moving window of 1,000 nucleotides, the pipeline excludes most SNPs present in horizontally transferred fragments of DNA that can introduce a number of SNPs in a single event, such as prophages, and mainly includes SNPs originating from point mutations that are transferred vertically. The resulting SNP matrix was then used to generate a maximum likelihood tree using RAxML, version 8.2.4 (75), with 100 bootstraps (-N 100) and the GTRCATX model (-m GTRCATX).

### Functional annotation of SNPs.

The program Variant Effect Predictor (VEP) (77) was used to annotate SNPs identified by the CFSAN SNP pipeline (76). Based on the genome annotation, variant call format (VCF) files were used to classify each SNP as (i) intergenic (falls within a noncoding region), (ii) synonymous (falls within a coding region but results in the same amino acid), (iii) nonsynonymous (falls within a coding region and results in an amino acid change), (iv) nonsense (falls within a coding region and introduces a premature stop codon), or (v) nonstop (changes a stop codon into an amino acid-encoding codon).

### Calculation of the estimated *dN*/*dS* per cluster.

The *dN*/*dS* value was calculated as follows: the number of nonsynonymous changes (Nc)/the number of nonsynonymous sites (Ns) divided by the number of synonymous changes (Sc)/the number of synonymous sites (Ss) (78). Thus, *dN*/*dS* is calculated as (Nc/Ns)/(Sc/Ss) = (Nc/Ns) × (Ss/Sc). A synonymous change is a nucleotide change in a protein-coding gene that does not change the amino acid encoded by the respective codon. Conversely, a nonsynonymous change is a nucleotide change in a protein-coding gene that also changes the amino acid encoded by the respective codon. A synonymous site is a nucleotide position in a protein-coding gene that could potentially be changed without changing the amino acid encoded by the respective codon. A nonsynonymous site is a nucleotide position in a protein-coding gene that could potentially be changed, and this change would result in a change in the amino acid encoded by the respective codon. The numbers of synonymous and nonsynonymous sites were empirically calculated using SNAP, version 2.1.1 (79), and the concatenated nucleotide sequences of the protein-coding genes present in the genome assemblies of the following isolates: (i) FSL N1-0013, representing cluster 1, (ii) FSL H1-0159, representing cluster 2, (iii) FSL T1-0027, representing subcluster 3a, and (iv) FSL T1-0077, representing subcluster 3b. The numbers of synonymous and nonsynonymous changes were calculated using all pairwise SNP differences observed within each cluster and subcluster.

### Tip-dated phylogenetic analysis.

The preserved SNPs identified by the CFSAN SNP pipeline were used to construct a tip-dated phylogeny of the cluster 3 isolates and to estimate the rate of evolution among these isolates. TempEst, version 1.5.1, was initially used to assess, using the hqSNP-based maximum likelihood tree, whether the data follow a single molecular clock (80). As the single molecular clock assumption could not be applied to our data, BEAUti (81) was used to create an XML file for the following models: (i) the relaxed clock log-normal clock model; (ii) the gamma site model using five gamma categories, shape estimated from the data (initial value of 1.0), proportion of invariant sites set at 0.0, and the standard K80 nucleotide substitution model; (iii) the coalescent Bayesian skyline tree prior model; (iv) the Jeffreys Markov chain population size prior model, with exponential gamma shape prior of 1.0 and offset prior of 0.0, log-normal mutation rate prior of 1.6 × 10^−7^ changes per nucleotide per year (estimated from Orsi et al. [2]), standard deviation prior of 0.2, and offset prior of 0.0. The Monte Carlo Markov chain was run 100,000,000 times and results (tracelog and treelog) were recorded every 10,000 runs. The relaxed clock log-normal clock model and the coalescent Bayesian skyline tree prior model were used as these models had been previously selected (using model selection by path sampling and calculation of Bayes factors) in a study of L. monocytogenes evolution using WGS SNP data (72). A separate XML file was created using the same settings but only the priors (no data) for comparison against the run with the empirical data. Ten replicate runs using the empirical data XML file were carried out using BEAST, version 2.5.2 (81), and the results from these 10 independent runs were combined using LogCombiner, version 2.5.1. Tree annotator, version 2.5.1, was used to sample the trees from the combined tree results using a 10% burn-in in order to obtain a maximum clade credibility tree with node height representing the common ancestor heights.

### Plasmid identification and annotation.

A total of 67 *Listeria* plasmids longer than 30,000 nucleotides were downloaded from NCBI on 25 October 2017. The plasmid sequences ranged from 32,307 to 89,986 nucleotides in length. These plasmids were further clustered using the program cd-hit, version 4.6.8 (82), with an overall alignment identity greater than 90% (i.e., -c 0.9). For each cluster, the one plasmid that showed the longest sequence was selected as representative and used for the subsequent analyses described here. Using this approach, 35 unique representative plasmids, ranging from 33,525 to 89,986 nucleotides in length, were selected (see Table S1 in the supplemental material) and used to create a BLAST nucleotide database (83) and an SRST2 database (84). The 42 draft genome assemblies were searched against the BLAST database using default blastn parameters with the exception of an E value cutoff of 1E−150 (i.e., -evalue 1e−150). The trimmed sequence paired reads for the 42 isolates were mapped against the SRST2 database using SRST2 with a minimum coverage of 90% (i.e., –min_coverage 90) and other default parameters. One representative of each plasmid identified in this study was further selected to analyze the phylogenetic relationship of the distinct plasmids. Plasmids were grouped based on clustering of the nucleotide sequences of the gene encoding the plasmid replication protein (primase protein). These sequences were aligned using ClustalW implemented in MEGA, version 7.0.18, with default parameters, and a maximum likelihood unrooted tree was constructed using RAxML (version 8.2.12) and the GTRCAT model. Clustering confidence was obtained by 1,000 bootstraps.

### Identification of stress response and virulence genes.

For the identification of single-nucleotide substitutions leading to premature stop codons in *inlA* and *prfA*, the full-length InlA and PrfA protein sequences from L. monocytogenes strain EGD-e were used to create a local protein BLAST database. This database was searched against the genome assemblies using blastx. The presence of an asterisk (*) in the matched query sequences was interpreted as the presence of a premature stop codon (PMSC) in the genome assembly sequences. In addition, for the identification of indels leading to premature stop codons in *inlA* and *prfA*, the nucleotide sequences for these two genes from L. monocytogenes strain EGD-e were used to create a local nucleotide BLAST database. This database was searched against the genome assemblies using blastn; if identified, indels were further assessed to determine whether they would cause a frameshift mutation leading to a PMSC in the sequence.

The nucleotide sequences for the individual genes present in the stress survival islets 1 and 2 (SSI-1 and SSI-2, respectively) were extracted from strains 10403S and CLIP11262, respectively, while the nucleotide sequences for the sanitizer tolerance genes *qacA*, *qacH* (*ermC*), and *emrE* were downloaded from the Pasteur MLST database (https://bigsdb.pasteur.fr/cgi-bin/bigsdb/bigsdb.pl?db=pubmlst_listeria_seqdef&page=downloadAlleles), and the nucleotide sequences for the sanitizer tolerance genes *qacE* (NCBI accession number NZ_AGUG01000015.1) and *bcrABC* (NCBI accession number JX023284.1) were downloaded from NCBI. All sequences were used to create a local nucleotide BLAST database. The database was searched against the draft assemblies using blastn and default parameters, and matches were translated into amino acid sequences to check for the presence of premature stop codons. Only matches with no premature stop codons and with more than 90% coverage and more than 90% identity were considered significant, and the genes were considered to be present in the respective isolate’s genome.

### Prophage sequence identification.

Prophage sequences were initially identified for each genome assembly using PHASTER (85). Only sequences classified by PHASTER as questionable (score 70 to 90) or intact (score of >90) were considered. Although unlikely, prophage sequences that were split into more than one contig may have been missed or partially predicted if none of the splits were classified as questionable or intact. Based on the PHASTER results, the prophage sequences identified were extracted from the genome assemblies and used to construct a local BLAST database. The database containing the prophage sequences was then searched against the genome assemblies to identify the following: (i) prophage sequences that were shared across multiple genomes (including sequences that were not identified by PHASTER as questionable or intact due to a split of the prophage sequence into multiple contigs) and (ii) prophage sequences that were unique to a given isolate. Prophage sequences were named ϕ-I to ϕ-XXII; prophage sequences received the same name if (i) the blastn match coverage was >95% of the subject sequence length and (ii) the nucleotide identity of the match was >99%. Presence of a prophage integrated into *comK* was assessed by using blastn to search for an intact *comK* sequence (609 nt) in each genome. Results with single matches of 609 nt were interpreted as indicating the absence of prophage integrated into *comK* (i.e., intact *comK*) while results with two matches (189 nt and 423 nt) were interpreted as indicating the presence of an integrated prophage into *comK*.

### Bacterial strain storage and cultivation.

A subset of five isolates, consisting of FSL H1-0506 (cluster 1), FSL M6-0204 (cluster 1), FSL H1-0322 (cluster 2), FSL T1-0027 (subcluster 3a), and FSL T1-0077 (subcluster 3b), was initially selected for the phenotypic experiments. In addition to these five isolates, seven other isolates, consisting of FSL R6-0670 (cluster 1), FSL H1-0159 (cluster 2), FSL L4-0166 (subcluster 3a), FSL R9-4443 (subcluster 3a), FSL T1-0938 (subcluster 3a), FSL N1-0053 (subcluster 3b), and FSL V1-0034 (subcluster 3b), were randomly selected from the remaining isolates from facility X after these remaining isolates were stratified by cluster, subcluster, and presence of a prophage sequence integrated into *comK*. These additional isolates resulted in a second subset with 12 isolates. Glycerol stock cultures in brain heart infusion (BHI) broth with 15% glycerol were stored at −20°C. For all phenotypic studies, cultures were streaked out on BHI agar plates, and plates were incubated for at least 30 h at 37°C. For all experiments except the attachment assay, a single colony from the incubated plate was transferred into 4 ml of BHI broth, followed by incubation (nonshaking) at 37°C for 12 to 16 h. After incubation, the culture was diluted 1:1,000 into 4 ml of prewarmed BHI broth, followed by incubation for 6.5 to 7 h at 37°C (nonshaking). For the attachment assay, a single colony from the incubated plate was transferred into 5 ml of BHI broth, followed by incubation (shaking) at 30°C for 12 to 16 h. This temperature was selected as previous studies have shown that flagella are involved in L. monocytogenes attachment to surfaces (64, 65) and that the expression of flagella is temperature dependent and occurs at 30°C or below (66, 67). After incubation, the cultures were diluted 1:100 into 5 ml of BHI broth, followed by incubation for 6.5 to 7 h at 30°C (shaking). The inoculum for the 96-well microtiter plate (which was used for attachment assays) was prepared by diluting 6.5- to 7-h cultures 1:100 in BHI broth to obtain ∼10^7^ CFU/ml. The inoculum for Bioscreen plates was prepared by diluting 6.5- to 7-h cultures in either BHI broth or modified BHI broth (for growth under different environmental stresses, e.g., low pH or high salt) to obtain ∼10^5^ CFU/ml. To enumerate the inoculum, dilutions of inoculum cultures were plated on BHI agar plates in duplicates, followed by incubation at 37°C for 24 h.

### Amplification of target genes on mobile elements.

The presence of selected genes (including *qacH*, *clpL*, and *bcrABC*) carried by plasmids or transposons was confirmed by PCR (primers are listed in Table 6). Colony lysates were prepared by using a 10-μl pipette tip to transfer part of a colony into a 0.2-ml reaction tube (Axygen, Union City, CA) containing 100 μl of double-distilled H_2_O (ddH_2_O). Reaction tubes were incubated at 98°C for 10 min in a Biometra thermocycler T-Gradient (Jena, Germany). The PCR master mix was prepared with Gotaq G2 (Promega, Duebendorf, Switzerland); 1 μl of the colony lysate was added into 14 μl of master mix. Samples were run in the thermocycler T-Gradient and then loaded on FlashGels for visualization of DNA amplification (Lonza, Basel, Switzerland) (Table S2 gives the master mix and thermocycler conditions).

**TABLE 6:** Primers used to target genes on mobile elements

Primer^*a*^	Target	Sequence 5′→3′	Annealing temp (°C)
clpL-F	*clpL*	GGATAATCAAAATTCGGAGCGTGC	56
clpL-R	*clpL*	TCATTCTCACGTCCAATCACTGG	
Tn6188qac-F	*qacH* on Tn*6188*	CACTTGCTTTATGATCAGGTTCTCC	56
Tn6188qac-R	*qacH* on Tn*6188*	GGGGGAAATTATTGGCTCTTCC	
bcrABC-F	*bcrABC*	CAAAAGGAGGGTAATCATGTCAGC	66^*b*^
bcrABC-R	*bcrABC*	GACAATTTAAGTACCACAACACCAGC	

a This study is the source for all primers.

b Twenty cycles with each cycle at −0.5°C and 20 final cycles at 56°C.

### Growth measurements with the Bioscreen.

Selected isolates were assessed for their growth (i) under low-QAC sanitizer concentrations and (ii) under different environmental stresses. Up to two 100-well honeycomb plates at a time were incubated in Bioscreen C (Oy Growth Curves Abs, Ltd., Helsinki, Finland) to measure the absorbance at the OD_600_ every 10 min. Measurements started within 10 min after loading of the instrument. Plates were incubated with continuous shaking using the medium setting. The absorbance values for cultures were determined by subtracting values for the medium blank from the values for the bacterial cultures at each time point; the detection threshold was set to an OD_600_ of 0.15. Three biological replicates for the sanitizer experiments, and the growth experiments under environmental stresses were performed on separate days, starting with colonies from different BHI agar plates; technical replicates originated from two individual colonies from the same agar plate.

### Growth in the presence of low sanitizer concentrations.

This experiment focused on QAC-based sanitizers as these sanitizers are among the most commonly used sanitizers in the food processing industry and as selected isolates contained QAC resistance genes. While concentrations used for these sanitizers are usually >200 mg/liter, low-level QAC concentrations were used here to be able to assess growth in the presence of these sanitizers and as previous data indicated that the identified QAC genes confer reduced sensitivity only to low levels of QACs. Four representatives of QAC sanitizers were selected for phenotypic characterization, including benzalkonium chloride (BC), benzethonium chloride (BZT), cetylpyridinium chloride (CPC), and Weiquat. Final concentrations used for growth experiments included the following: (i) BC at 1, 2, 3, 4, and 5 mg/liter, (ii) BZT at 1, 2, 3, 4, 5, 6, and 7 mg/liter, (iii) CPC at 1, 2, 3, 4, and 5 mg/liter, and (iv) Weiquat at 0.001%, 0.002%, 0.003%, 0.004%, and 0.005%. Sanitizers were prepared in BHI broth, and 200 μl of BHI with sanitizer was loaded into each well of 100-well honeycomb plates. A 200-μl inoculum was added into each well, and honeycomb plates were incubated in a Bioscreen at 22°C for 24 h, with shaking. The MIC was defined as the lowest sanitizer concentration at which the detection threshold of an OD_600_ of 0.15 was not reached by all biological replicates after 24 h. The subset of five isolates was used for the initial assessment of growth in the presence of all four sanitizers, while the subset of 12 isolates was used to assess the growth in the presence of BC.

### Growth under different environmental conditions.

Growth of the initial subset of five isolates was assessed in BHI broth at 37°C (nonstress) as well as under selected stress conditions (defined as deviating from optimal growth conditions for *Listeria*, which are 37°C, pH 7.0, and an a_w_ of 0.995 [86]), including the following: (i) BHI broth with incubation at 15.5°C (minimum temperature that could be achieved with the instrument) and 40°C; (ii) BHI broth with reduced water activity of 0.95, achieved by addition of 15.6% (vol/vol) glycerol; (iii) BHI broth with pH adjusted to 5.5 by addition of hydrochloric acid; and (iv) BHI broth with addition of 6% NaCl (6.5% NaCl final concentration). Honeycomb plates were preloaded with 200 μl of BHI broth or modified BHI broth, and 200 μl of inoculum (in BHI broth or the appropriate modified BHI broth) was added into the first well, followed by 2-fold dilutions to achieve a total of eight dilutions. Plates were incubated in the Bioscreen at 37°C unless a different temperature is stated. Plates were incubated until all dilutions reached stationary phase (based on optical density growth curve plots).

### Survival under oxidative stress.

The subset of five selected isolates was also assessed for oxidative stress survival. After 6.5 to 7 h of growth, bacterial cultures were adjusted to an OD of 0.2 (Libra S4 spectrophotometer; Biochrom Bioswisstec AG, Schaffhausen, Switzerland) with defined minimal medium containing 10 mM glucose (87). OD-adjusted cultures were diluted 1:2 in defined minimal medium in three 50-ml Falcon tubes with a final volume of 10 ml containing either 10 mM cumene hydrogen peroxide (CUHP) dissolved in dimethyl sulfoxide (DMSO) or DMSO only, in addition to a control without treatment. After 2 h of incubation at 37°C, 1 ml of culture was spun down at 12,000 rpm for 2 min, the supernatant was removed, and the pellet was resuspended in 1 ml of defined minimal medium; this washing step was repeated twice. Dilutions of washed cells were plated on BHI agar, followed by incubation for 48 h at 37°C.

### Attachment assay in 96-well microtiter plates.

Attachment of the subset of 12 isolates was assessed in a polystyrene 96-well microtiter plate by crystal violet staining. The Pseudomonas aeruginosa ATCC 10145 (FSL R8-4871) strain, which has been previously shown to form biofilm (88), was included as a positive control. Attachment of isolates was assessed as previously described by Lourenco et al. (89). In brief, 96-well polystyrene plates were loaded with 150 μl of bacterial cultures (∼10^7^ CFU/ml) and wrapped with parafilm, followed by incubation (nonshaking) for 120 h at 10°C or 21°C. After incubation, the supernatant was removed, and wells were washed three times with 200 μl of dH_2_O, followed by drying in a biosafety hood for 30 min. Remaining cells in the 96-well plates were stained by addition of 50 μl of 0.35% (wt/vol) crystal violet solution, followed by incubation at room temperature in a biosafety hood for 45 min. The staining solution was removed, and wells were washed three times with 200 μl of dH_2_O. In order to solubilize the stain, 200 μl of 95% ethanol (vol/vol) was added to each well, and plates were incubated at 4°C for 30 min. Following incubation, 100 μl was transferred into new wells, absorbance was measured at an OD_600_ with a Synergy Reader H1 (BioTek, VT, USA), and values obtained from wells previously filled with uninoculated BHI medium were subtracted.

### Statistical analysis.

Data were analyzed in R, version 3.4.0 (R Core Team, Vienna, Austria). A two-sided Fisher’s exact test was used to assess associations of presence of QAC sanitizer tolerance genes and stress survival islets with persistent (isolated over more than a 1-year period) and transient (isolated over less than a 1-year period) isolates. Based on growth curve data generated under various stresses, a logistic equation was fitted using the SummarizeGrowth function in the growthcurver package, version 0.3.0 (90), to obtain the growth rate and the absorbance at stationary phase. A linear mixed-regression model was fitted to each growth curve parameter using the package lme4, version 1.1-13 (91); isolate, condition, and isolate-condition interaction were included as fixed effects while biological replicates and dilution were included as random effects. Statistical analyses were performed using emmeans, version 1.2.3 (92), and *post hoc* multiple-comparison adjustment was performed with Tukey’s honestly significant differences (HSD) test. For the statistical analysis of the attachment assay, four linear mixed-effects models were fitted to absorbance (response variable) using “replicates” as a random variable; these four linear mixed effects models included the fixed predictor variable “temperature,” as well as the following: (i) “isolate” and “isolate-temperature interaction,” (ii) “cluster and subcluster” and “cluster and subcluster-temperature interaction,” (iii) “persistent or transient” and “persistent or transient-temperature interaction,” and (iv) “*comK* or phage” (where “*comK*” indicates an intact *comK* sequence and “phage” indicates a *comK* sequence interrupted by a prophage sequence since a *comK* sequence interrupted by a prophage had previously been linked to enhanced attachment in L. monocytogenes [11]) and “*comK* or phage-temperature interaction.” Additional linear mixed-effects models were fitted to absorbance using “replicates” as a random variable and including the fixed predictor variables (i) “cluster and subcluster” and (ii) “isolate” at each of the two temperatures (10°C and 21°C), followed by statistical analysis using emmeans and *post hoc* multiple-comparison adjustment performed with Tukey’s HSD test. All experiments were conducted in three biological replicates with two technical replicates, results of which were averaged, except for oxidative stress experiments, which were performed with one technical replicate. Adjusted *P* values of ≤0.05 were considered statistically significant.

## Supplementary Material

Supplemental file 1
