# Supplementary material for: Evolution of Listeria monocytogenes in a Food Processing Plant Involves Limited Single-Nucleotide Substitutions but Considerable Diversification by Gain and Loss of Prophages
Source: Appl Environ Microbiol. 2020 Mar 2;86(6):e02493-19. doi: 10.1128/AEM.02493-19 (PMC7054086; doi:10.1128/AEM.02493-19)
Supplement: Supplemental file 1 [file AEM.02493-19-s0001.pdf]

## SUPPLEMENTAL FIGURE LEGENDS

**Supplemental Figure SF1.** Gene tree based on the primase gene sequences extracted from the plasmids found in FSL N1-0013 (cluster 1), FSL H1-0322 (cluster 2), FSL H6-0175 (sub-cluster 3a), FSL T1-0077 (sub-cluster 3b) and FSL R9-4003 (unclustered isolate). Bootstrap values (1,000 replicates) are shown next to nodes. The branch between groups A and B and group C was shortened for display. The horizontal line provides the scale for the estimated genetic distance. Groups A, B and C are labelled (See Table 2).

**Supplemental Figure SF2.** Time to detection in hours (over 24 h of incubation at 22°C) (y-axis) for *Listeria* strains (x-axis) in absence of sanitizer (0 mg/L for BC, BZT, CPC or 0% for Weiquat) and in presence of (A) BC from 1 mg/L to 3 mg/L – no growth was detected at 4mg/L or 5mg/L, (B) BZT from 1 mg/L to 6 mg/L – no growth was detected at 7mg/L, (C) CPC from 1 mg/L to 3 mg/L – no growth was detected at 4 mg/L or 5mg/L, (D) Weiquat from 0.001% to 0.003% - no growth was detected at 0.004% or 0.005%. Solid line indicates limit of incubation time at 24 h. The sanitizer concentrations are color coded and each biological replicate at each concentration is represented by a dot (n = 3). The midline of the boxplot represents the median, the lower and upper limit of the box represent the 25$^{th}$ and 75$^{th}$ percentile, respectively.

**Supplemental Figure SF3.** Time to detection in hours (over 24 h of incubation at 22°C) (y-axis) for *Listeria* strains (x-axis) in absence of sanitizer (0 mg/L) and in presence of BC from 1 mg/L to 3 mg/L – no growth was detected at 4mg/L or 5mg/L. Solid line indicates limit of incubation time at 24 h. The sanitizer concentrations are color coded and each biological replicate at each concentration is represented by a dot (n = 3). The midline of the boxplot represents the median, the lower and upper limit of the box represent the 25$^{th}$ and 75$^{th}$ percentile, respectively.

**Supplemental Figure SF4.** Estimated marginal means of (A) growth rate in generations per hour, and (B) OD at early stationary phase for each *Listeria* strain (x-axis) when grown in different conditions, including 37°C (37C), 15°C (15C), 40°C (40C), reduced water activity 0.95 (aw), additional 6% NaCl (NaCl), and pH 5.5 (pH5.5) displayed in each grid. Colors indicate the assigned sub-cluster based on phylogenetic analysis. The letter code is based on post-hoc Tukey analysis; dots that do not share the same letter (within a given box) are significantly different. The bars indicate the estimated upper and lower confidence interval at 95% based on the linear mixed regression model with three biological replicates.

**Supplemental Figure SF5.** Estimated means of log reduction (y-axis) for each strain (x-axis) after exposure to 10 mM CUHP for 2 h. Error bars indicate the standard error of three biological replicates. ANOVA found no evidence for significant difference in log reduction among the strains.

**Supplemental Figure SF6. Attachment by individual strains.** Arithmetic mean of absorbance measured at $OD_{600nm}$ by individual isolates (x-axis) at (A) 10°C and (B) 21°C. Colors and small numbers above the bar indicate the assigned cluster and sub-cluster based on phylogenetic analysis. The letter code is based on post-hoc Tukey analysis; bars that do not share the same letter (within a given incubation temperature) are significantly different. The bars indicate the standard deviation based on three biological replicates.

Supplemental Figure SF1

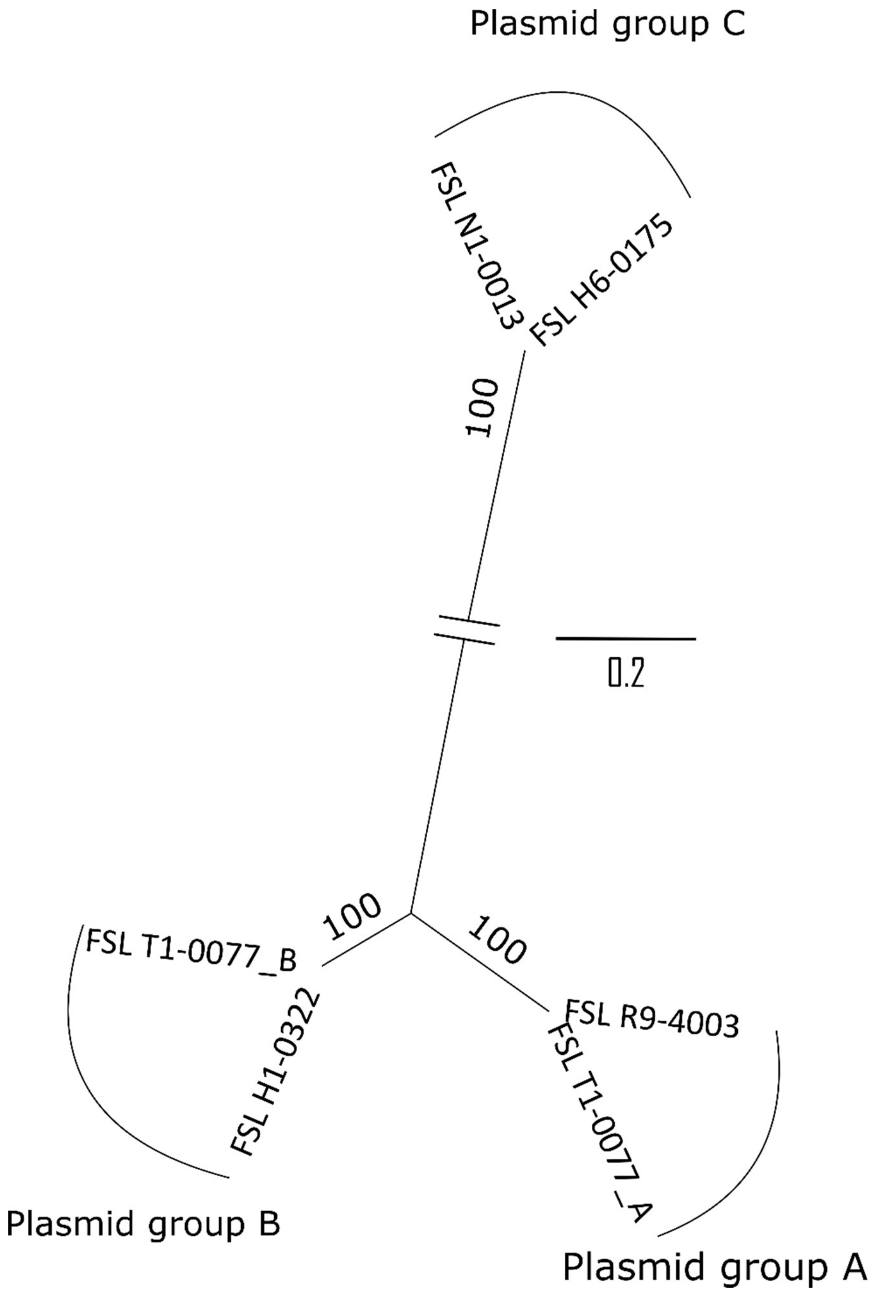

## Supplemental Figure SF2

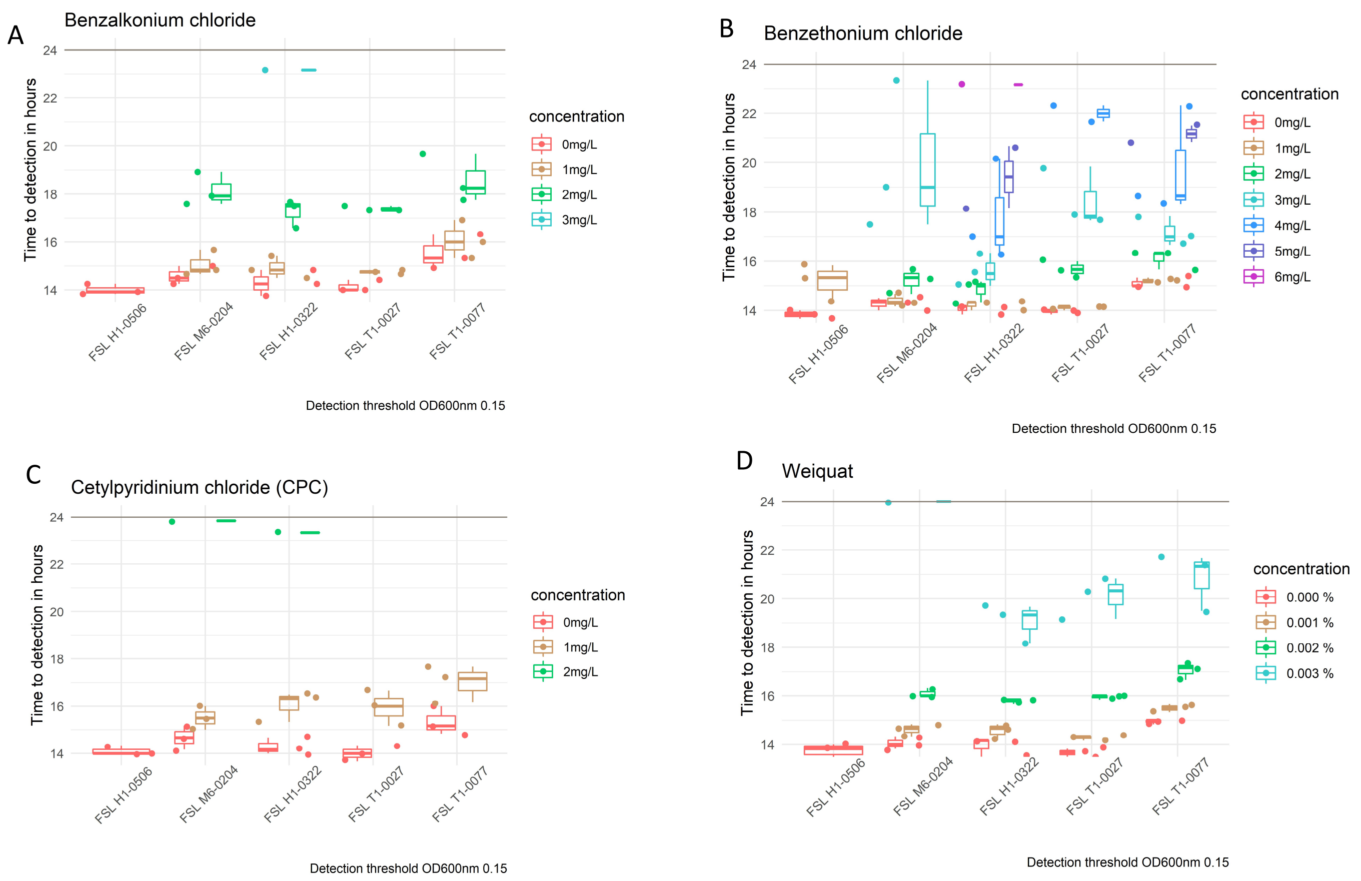

Supplemental Figure SF3

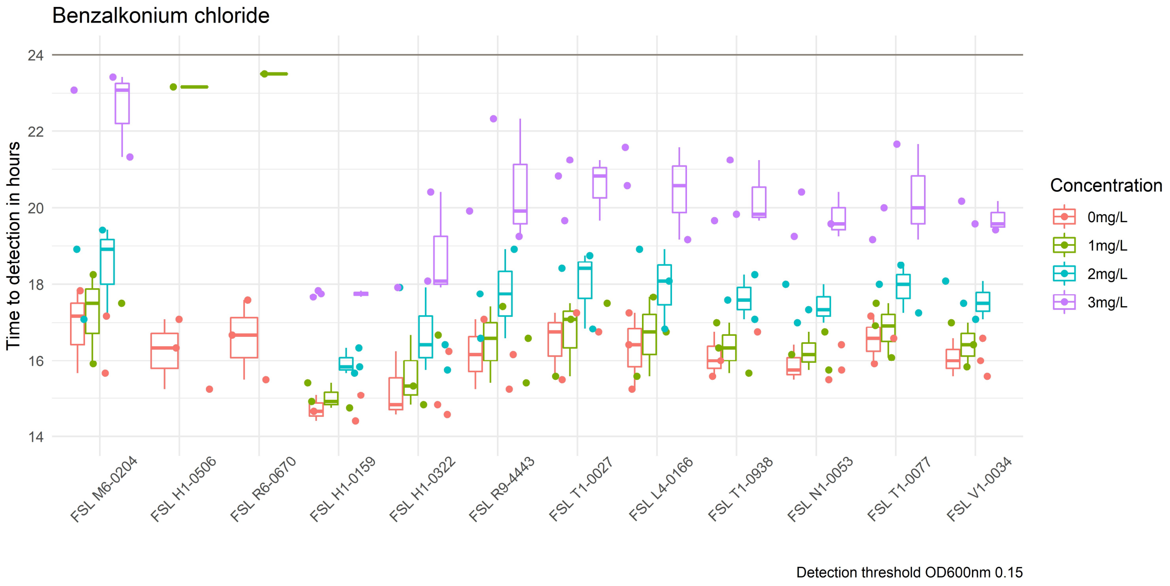

Supplemental Figure SF4

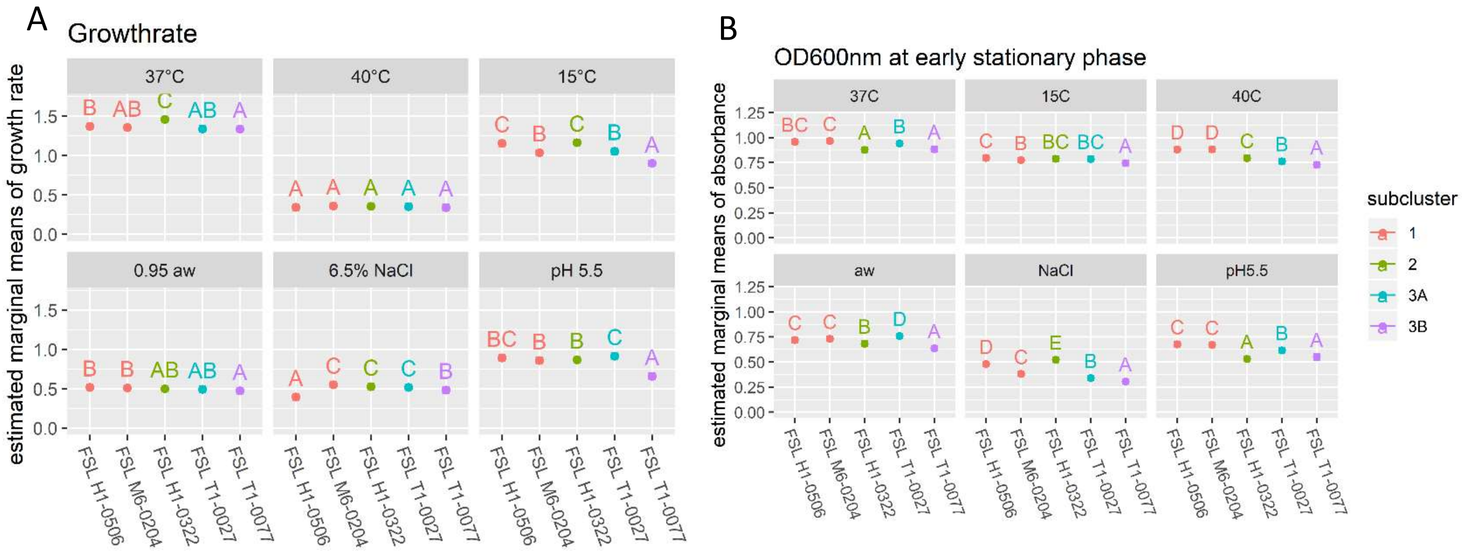

Supplemental Figure SF5

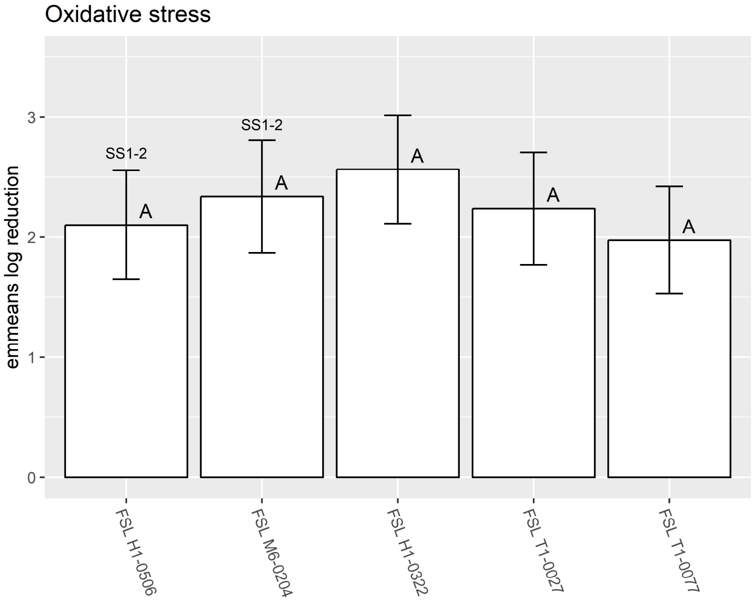

Supplemental Figure SF6

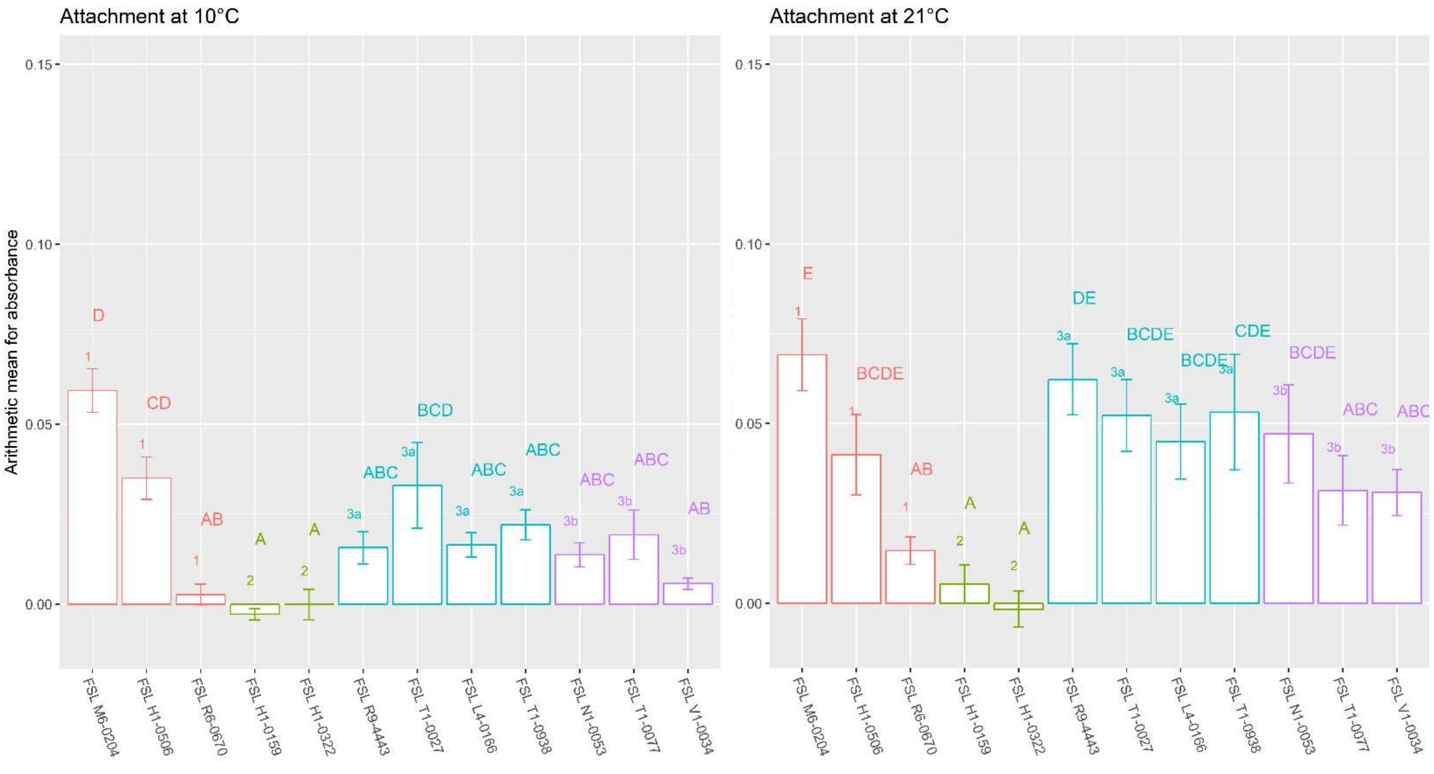

**Supplemental Table ST1. NCBI Nucleotide database accession numbers for the 35 plasmid sequences used to create the BLAST and SRST2 nucleotide databases**

| Accession |
|---|
| NC_003383.1 |
| NC_010893.1 |
| NC_011101.1 |
| NC_013767.1 |
| NC_014495.1 |
| NC_014496.1 |
| NC_015148.1 |
| NC_018888.1 |
| NC_021828.1 |
| NC_021871.1 |
| NC_022046.1 |
| NZ_CM001470.1 |
| NZ_CM008329.1 |
| NZ_CP011399.1 |

| |
|---|
| NZ_CP013725.1 |
| NZ_CP014251.1 |
| NZ_CP014254.1 |
| NZ_CP015985.1 |
| NZ_CP019168.1 |
| NZ_CP020829.1 |
| NZ_CP020834.1 |
| NZ_CP023051.1 |
| NZ_CP023053.1 |
| NZ_CP023753.1 |
| NZ_GL538355.1 |
| NZ_HG813248.1 |
| NZ_JZBQ01000031.1 |
| NZ_JZCS01000018.1 |
| NZ_JZCS01000019.1 |
| NZ_JZCT01000028.1 |
| NZ_JZCU01000022.1 |

| |
|---|
| NZ_JZHC01000043.1 |
| NZ_LXQP01000010.1 |
| NZ_MBOL01000016.1 |
| NZ_MCHQ01000007.1 |

**Supplementary Table ST2.** Mastermix preparation

| Reagent | Volume in uL |
|---|---|
| Nuclease-free H2O | 6 |
| 2x Gotaq G2 buffer | 7.5 |
| 10uM Primer F | 0.75 |
| 10uM Primer R | 0.75 |

**Supplemental Table ST3.** Model parameters for linear mixed effect model, assessing the effect of individual isolates on attachment of selected *L. monocytogenes* isolates at 10°C and 21°C

| Response variable | Fixed Effects | Chiseq[1] | Df[1] | P-value[1] | Significance[1] |
|---|---|---|---|---|---|
| Absorbance | | | | | |
| | Temperature | 43.315 | 1 | <0.001 | *** |
| | Isolate | 151.367 | 11 | <0.001 | *** |
| | Temperature*Isolate | 21.015 | 11 | 0.033 | * |

[1] Based on analysis of deviance for type II wald chisquare test, parameters only available for explanatory variables from model.

***$P < 0.001$; **$P < 0.01$; *$P < 0.05$

**Supplemental Table ST4.** Model parameters for linear mixed effect model, assessing the effect of cluster and sub-cluster on attachment of selected *L. monocytogenes* isolates at 10°C and 21°C

| Response variable | Fixed Effects | Chiseq[1] | Df[1] | P-value[1] | Significance[1] |
|---|---|---|---|---|---|
| Absorbance | | | | | |
| | Temperature | 22.233 | 1 | <0.001 | *** |
| | Cluster_subcluster | 43.399 | 3 | <0.001 | *** |
| | Temperature*Cluster_subcluster | 7.674 | 3 | 0.053 | |

[1] Based on analysis of deviance for type II wald chisquare test, parameters only available for explanatory variables from model.

***$P < 0.001$; **$P < 0.01$; *$P < 0.05$

**Supplemental Table ST5.** Model parameters for linear mixed effect model, assessing the effect of persistence on attachment of selected *L. monocytogenes* isolates at 10°C and 21°C

| Response variable | Fixed Effects | Chiseq[1] | Df[1] | P-value[1] | Significance[1] |
|---|---|---|---|---|---|
| Absorbance | | | | | |
| | Temperature | 14.241 | 1 | < 0.001 | *** |
| | Persistent_transient[2] | 2.758 | 1 | 0.097 | |
| | Temperature*Persistent_transient[2] | 4.373 | 1 | 0.037 | * |

[1] Based on analysis of deviance for type II wald chisquare test, parameters only available for explenatory variables from model.

[2] Refers to strains being either persistent (isolated over more than a 1-year period) or transient (isolated over less than a 1-year period)

****P* < 0.001; ***P* < 0.01; **P* < 0.05

**Supplemental Table ST6.** Model parameters for linear mixed effect model, assessing the effect of phage integration into *comK* on attachment of selected *L. monocytogenes* isolates at 10°C and 21°C

| Response variable | Fixed Effects | Chiseq[1] | Df[1] | P-value[1] | Significance[1] |
|---|---|---|---|---|---|
| Absorbance | | | | | |
| | Temperature | 13.104 | 1 | < 0.001 | *** |
| | Phage_*comK*[2] | 0.072 | 1 | 0.788 | |
| | Temperature*Phage_*comK*[2] | 1.363 | 1 | 0.243 | |

[1] Based on analysis of deviance for type II wald chisquare test, parameters only available for explenatory variables from model.

[2] Phage_*comK* refers to isolates with prophage integration in *comK* ("Phage") or with intact *comK* gene ("*comK*")

***$P < 0.001$; **$P < 0.01$; *$P < 0.05$
